# Metabolic cell deaths in head and neck cancer: mechanisms, therapeutic potential, and challenges

**DOI:** 10.1080/07853890.2025.2582906

**Published:** 2025-11-04

**Authors:** Tong Wang, Xu Li, Xu Huang, Zhidong Wang, Yangchun Zhang, Junfang Lei, Xinrui Qian, Chang Lu, Zhaohui Liu

**Affiliations:** Department of Otorhinolaryngology, Affiliated Hospital of Zunyi Medical University, Zunyi, China

**Keywords:** Head and neck cancer (HNC), ferroptosis, cuproptosis, metabolic cell death, therapeutic target

## Abstract

**Background:**

Head and neck cancer (HNC) poses prominent clinical challenges: limited early diagnosis efficacy, suboptimal five-year survival after conventional treatment, over 60% of patients at advanced/metastatic stages, and lymph node metastasis accelerating progression. Metabolic cell death (ferroptosis, cuproptosis, disulfidptosis, lysozincrosis, alkaliptosis) offers promising HNC therapeutic avenues, while their crosstalk, synergy with standard therapies and translation hurdles require attention.

**Main findings:**

Ferroptosis inducers demonstrate capacity to reverse HNC drug resistance and synergize with chemoradiotherapy, yet elevated FSP1 expression drives resistance. Cuproptosis inducers combined with immunotherapy reprogram the tumor microenvironment, with HIF-1α mediating resistance to this combination. HNC exhibits sensitivity to disulfidptosis, but its *in vivo*/*in vitro* validation in HNC remains insufficient. Lysozincrosis, triggered by lysosomal zinc release, has TRPML1 as a potential target due to HNC’s active lysosomal function. Alkaliptosis induces selective cancer cell death and is regulated by CA9 and NF-κB. These pathways interact, and their translation is hindered by tumor heterogeneity and poor delivery efficiency.

**Future directions:**

Future studies should prioritize clarifying these pathways’ regulatory network, address translation hurdles, develop multi-target strategies and tumor-targeted delivery systems, and enhance therapy synergy to improve HNC prognosis.

## 1. Introduction

In 2022, head and neck cancer (HNC) was recorded as the sixth most common cancer globally [1]. This type of cancer originates from the epithelial cells found in the mucosal tissues of the oral cavity, pharynx, and larynx, making it the most frequently occurring malignant tumor in the head and neck region [2]. The primary risk factors associated with HNC include tobacco use, alcohol consumption, betel quid chewing, and human papillomavirus (HPV) infection [3]. More than 60% of patients with HNC are diagnosed at an advanced or metastatic stage of the disease [4]. Treatment modalities for HNC primarily consist of surgery, radiotherapy, and chemotherapy. For early-stage HNC, surgery or radiotherapy is typically the preferred treatment. In cases of advanced or recurrent disease, a more comprehensive treatment strategy is generally employed [5]. However, the 5-year overall survival rate for HNC remains low following conventional treatments [6]. Therefore, identifying innovative therapeutic strategies is particularly crucial for individuals diagnosed with advanced or recurrent HNC, as this could significantly improve their outcomes.

Lymph node metastasis (LNM) is a critical event in the progression of HNC and serves as one of the strongest prognostic indicators, closely associated with poorer overall survival and an increased risk of distant metastasis [7]. In addition to the anatomical burden, tumor-draining lymph nodes experience immune remodeling that fosters tolerance, potentially facilitating systemic dissemination. Although pathology remains the diagnostic gold standard, the early and accurate identification of microscopic nodal disease presents significant challenges. Multimodal imaging, along with sentinel lymph node biopsy where appropriate, can enhance staging and guide comprehensive treatment strategies. Considering the prognostic significance of nodal status and its interaction with host immunity, therapeutic approaches that target metabolic cell death pathways—such as ferroptosis, cuproptosis, and disulfidptosis—may affect both primary tumor sensitivity and nodal biology. Furthermore, these approaches could synergize with immuno-oncology to improve outcomes in patients with node-positive or recurrent/metastatic HNC.

Metabolic cell death is defined as the demise of cells resulting from either an excess or deficiency of specific nutrients or metals within the cellular environment, leading to an imbalance in metabolic processes. A comparable form of cell death is cellular suicide, a regulated process directly initiated by signaling pathways and proteins that execute cell death, aimed at eliminating unwanted cells within the organism [8]. Common examples of metabolic cell death include ferroptosis, cuproptosis, disulfidptosis, lysozincrosis, and alkaliptosis.

In this review, we emphasize that individuals diagnosed with HNC face a dismal prognosis, as conventional treatment modalities often prove inadequate for certain metastatic and recurrent cases. Therefore, there is an imperative need to explore novel treatment methodologies and frameworks to enhance standard therapies, which could ultimately improve outcomes for these patients. This has sparked our interest in the concept of metabolic cell death. Our research aims to investigate the intricate mechanisms underlying metabolic cell death, analyze several newly identified pathways, including ferroptosis, cuproptosis, disulfidptosis, lysosomal zinc-induced cell death, and alkaliptosis, and evaluate their therapeutic potential, as well as the challenges they present in the context of HNC. For the first time, this study systematically compares the cross-regulation of ferroptosis, cuproptosis, and disulfidptosis in HNC, thereby addressing a significant research gap in this field.

## 2. The mechanism and research progress of ferroptosis in HNC

Ferroptosis, identified by Dixon et al. [9] in 2012 as a distinct form of regulated cell death (RCD), is characterized by an iron-dependent mechanism resulting from the overaccumulation of lipid peroxides in cellular membranes. Research surrounding ferroptosis has increasingly taken center stage in the healthcare arena, especially in the field of oncology. Ferroptosis inducers show considerable promise in clinical treatment. For tumors that demonstrate escalating resistance to chemotherapy, including liver cancer, advanced gastric cancer, and HNC, employing ferroptosis inducers as an innovative therapeutic approach, customized to the distinct features of ferroptosis in tumor cells, has the potential to lead to enhanced clinical results.

### 2.1. Molecular biological basis of ferroptosis

#### 2.1.1. Iron metabolism and lipid peroxidation

Iron is taken up in the upper jejunum and duodenum, originating from dietary sources, heme iron released by macrophages, as well as iron reserves found in hepatocytes [10]. It is taken up by intestinal epithelial cells in its divalent state or bound to transport proteins, undergoing a series of processes to enter the bloodstream and be distributed to various organs. When iron levels are low, the body regulates iron homeostasis via selective autophagy degradation and releases ferritin to replenish iron stores [11]. Intracellular iron levels are influenced by various factors, including autophagy, which modulates ferritin degradation and iron release through a process termed ‘ferritinophagy’ [12]. Cell membranes in mammals are abundant in phospholipids that include polyunsaturated fatty acids (PUFAs) [13], rendering them especially susceptible to lipid peroxidation [14]. The primary mechanism driving membrane oxidation during ferroptosis is non-enzymatic lipid peroxidation induced by Fenton-like reactions [15,16]. Lipoxygenases (LOXs) are involved in this process. Products of lipid peroxidation, including lipid hydroperoxides (PLOOHs), may damage the integrity of cell membranes and hinder cellular functions. Additionally, these products can regulate cell death processes through various mechanisms [17].

#### 2.1.2. Antioxidant defense systems and ferroptosis

The primary intracellular endogenous antioxidant systems consist of enzymatic and non-enzymatic antioxidants [18,19]. Glutathione peroxidase (GPx) consists of several isoenzymes, each characterized by different localizations within cells and unique expression patterns that vary by tissue [20,21]. Glutathione peroxidase 4 (GPX4), also known by its alternative name phospholipid hydroperoxide glutathione peroxidase (PHGPx) [22], is recognized as the fourth enzyme within the GPX family. This enzyme incorporates selenium as a crucial component in its structure and function. This enzyme is crucial for cellular ferroptosis and uniquely functions as a phospholipid hydroperoxidase within the GPX family. It directly transforms PLOOH into its related phospholipid alcohol (PLOH), which is crucial for protection against lipid peroxidation [23,24]. The GPX4 protein facilitates the clearance of lipid peroxides; its inactivation disrupts oxidative balance, leading to lipid peroxides damaging cell membrane structures and activating ferroptosis [25]. Glutathione (GSH) serves as a cofactor for several antioxidant enzymes, with a notable emphasis on GPX4 [26]. The catalysis of GPX4 is contingent upon the availability of reduced glutathione (GSH), which necessitates a continuous supply of cystine. In human cells, the uptake of cystine is facilitated by System Xc^-^, where the light-chain subunit xCT is encoded by the SLC7A11 gene. This connection establishes a link between SLC7A11 and the functional dynamics of System Xc^-^, as well as the SLC7A11–GPX4 axis [27]. Consequently, the inhibition of GPX4 or the disruption of cystine supply—such as the blockade of System Xc^-^ or the depletion of GSH—results in the accumulation of PLOOH. In the presence of iron, PLOOH generates lipid radicals, thereby triggering ferroptosis [25].

**Figure 1.**
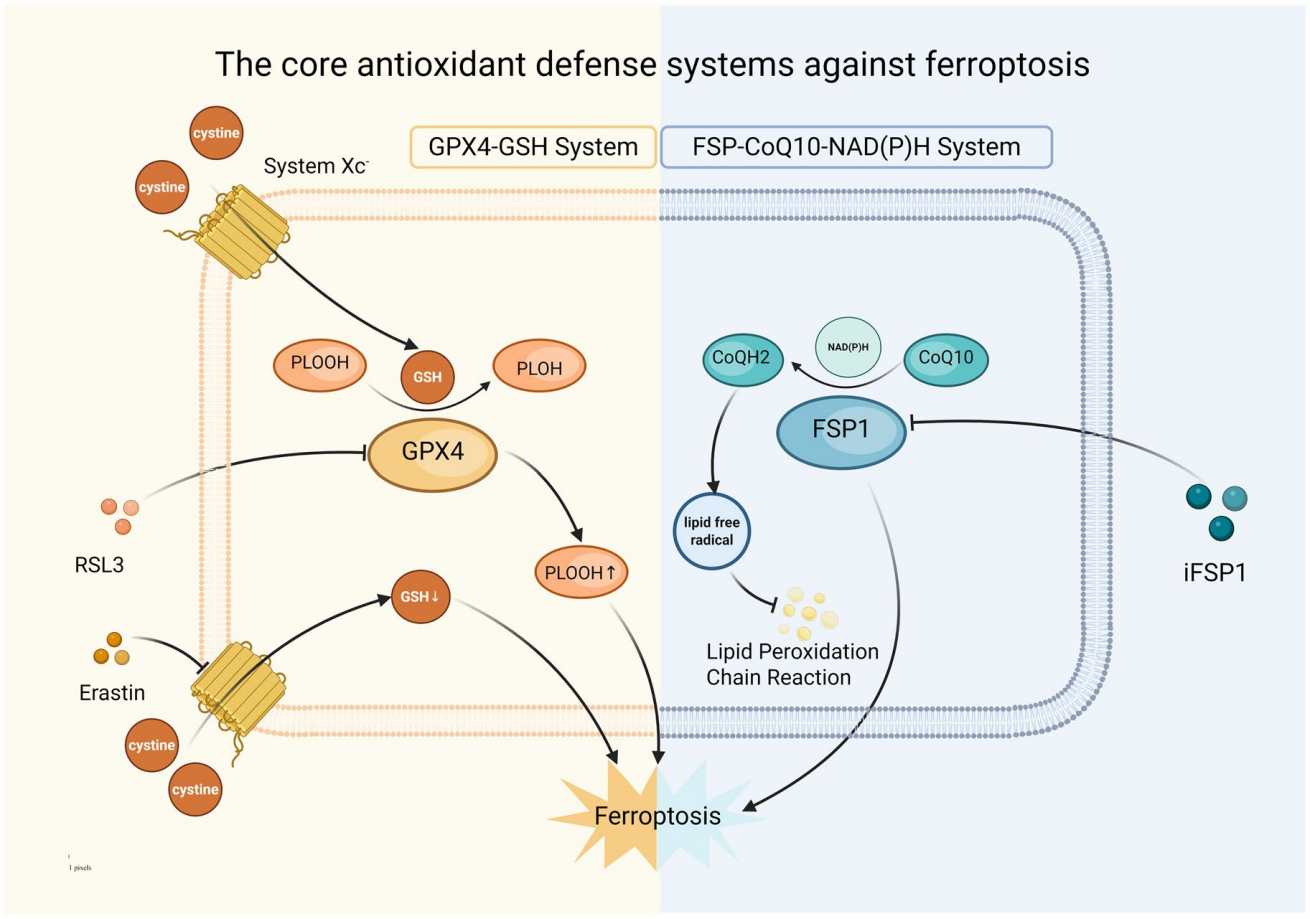
The figure illustrates the two core antioxidant defense systems of cells against ferroptosis. The left system is the GPX4-GSH system, wherein System Xc^-^ mediates the cellular uptake of cystine, providing the necessary materials for GSH synthesis. GSH acts as a cofactor for GPX4, assisting in the reduction of PLOOH to PLOH, thereby clearing lipid peroxides, maintaining oxidative homeostasis, and inhibiting ferroptosis. When System Xc^-^ is blocked by Erastin, GPX4 is inhibited by RSL3, or GSH is depleted, PLOOH accumulates significantly, triggering ferroptosis. The right system is the FSP1-CoQ10-NAD(P)H system, where FSP1 utilizes NAD(P)H as an electron donor to catalyze the reduction of CoQ10 to ubiquinol CoQH_2_. CoQH_2_ can scavenge lipid free radicals, block the lipid peroxidation chain reaction, and exert an anti-ferroptotic effect. This system operates in parallel and independently of the GPX4-GSH system; even if GPX4 is inactivated, FSP1 can still inhibit lipid peroxidation at the cell membrane through this pathway. Additionally, the inhibitor iFSP1 can act on FSP1 to promote the occurrence of ferroptosis.

Ferroptosis suppressor protein 1 (FSP1, also known as AIFM2) is a FAD/NAD(P)H-dependent oxidoreductase that localizes to the cell membrane via N-terminal myristoylation. It catalyzes the reduction of ubiquinone (CoQ_10_) to ubiquinol (CoQH_2_), scavenging lipid free radicals and thereby blocking the propagation of lipid peroxidation chains to exert anti-ferroptotic effects [28,29]. The FSP1–CoQ_10_–NAD(P)H axis functions as a parallel and independent defense mechanism to the GPX4–GSH system. Notably, even in the context of GPX4 inactivation, FSP1 can still significantly inhibit lipid peroxidation at the cell membrane, establishing a dual-layered protective system [30–33]. FSP1 is subject to complex regulatory mechanisms, including transcriptional control by factors such as p53, which represses AIFM2 gene expression, and modulation via the NRF2–KEAP1 axis. Additionally, FSP1 is regulated by non-coding RNAs, as well as post-translational modifications such as myristoylation, which governs its subcellular localization and enzymatic activity [28,29,34]. Studies have demonstrated elevated expression of FSP1 in samples from patients with recurrent or cisplatin-resistant HNC. Such high expression is closely associated with the emergence of drug-tolerant persisters (DTPs), increased cancer cell stemness, enhanced invasion and metastasis, and overall therapeutic resistance. Knockdown or pharmacological inhibition of FSP1 (e.g. via iFSP1) significantly enhances chemosensitivity, suppresses stemness and invasiveness, and effectively promotes ferroptosis in patient-derived models [35] (Figure 1).

### 2.2. The relationship between ferroptosis and HNC

#### 2.2.1. Impact on the proliferation and survival of HNC cells

HNC cells exhibit dysregulated iron metabolism, characterized by elevated labile iron pools, increased transferrin receptor (TfR) expression, and altered iron regulatory protein (IRP) function—especially IRP2—creating an iron-rich microenvironment that supports DNA replication, oxidative stress resistance, and metastasis [36,37]. Early ferroptosis in tumor cells releases damage-associated molecular patterns (DAMPs), which can activate dendritic cell-mediated anti-tumor immunity but also promote pro-inflammatory tumor progression [38,39]. HNC’s mesenchymal phenotype and lipid peroxidase pathway dependence enhance sensitivity to GPX4 inhibition, with GPX4 blockade inducing ferroptosis to suppress cell proliferation *via* SREBP pathway downregulation [40,41].

Notably, in cisplatin-treated and recurrent HNSCC, the expression of FSP1 is significantly upregulated, allowing tumor cells to evade ferroptosis. Targeting FSP1, either through pharmacological inhibition or genetic knockdown, can reverse this resistance, increase ferroptotic susceptibility, and enhance the chemosensitivity of cancer cells [42]. Moreover, FSP1 operates independently of the GPX4–GSH system, suggesting that co-targeting FSP1 and GPX4 may synergistically trigger ferroptosis and circumvent tumor cell survival mechanisms. These findings underscore the therapeutic potential of FSP1, particularly in the context of cisplatin-resistant tumors and drug-tolerant cancer cell subpopulations. Ferroptosis inducers like Erlotinib increase reactive oxygen species (ROS) to inhibit tumor growth, while CISD2 inhibition overcomes azathioprine resistance [43,44]. HPV-positive HNC cells, with hyperactive iron metabolism, are more prone to ferroptosis, whereas HPV-negative subtypes favor apoptosis [45]. ACSL4/FSP1/PCBP1 constitutes the HNC ferroptosis regulatory axis, among which the FSP1-CoQ10 pathway mediates primary drug resistance [46].

**Figure 2.**
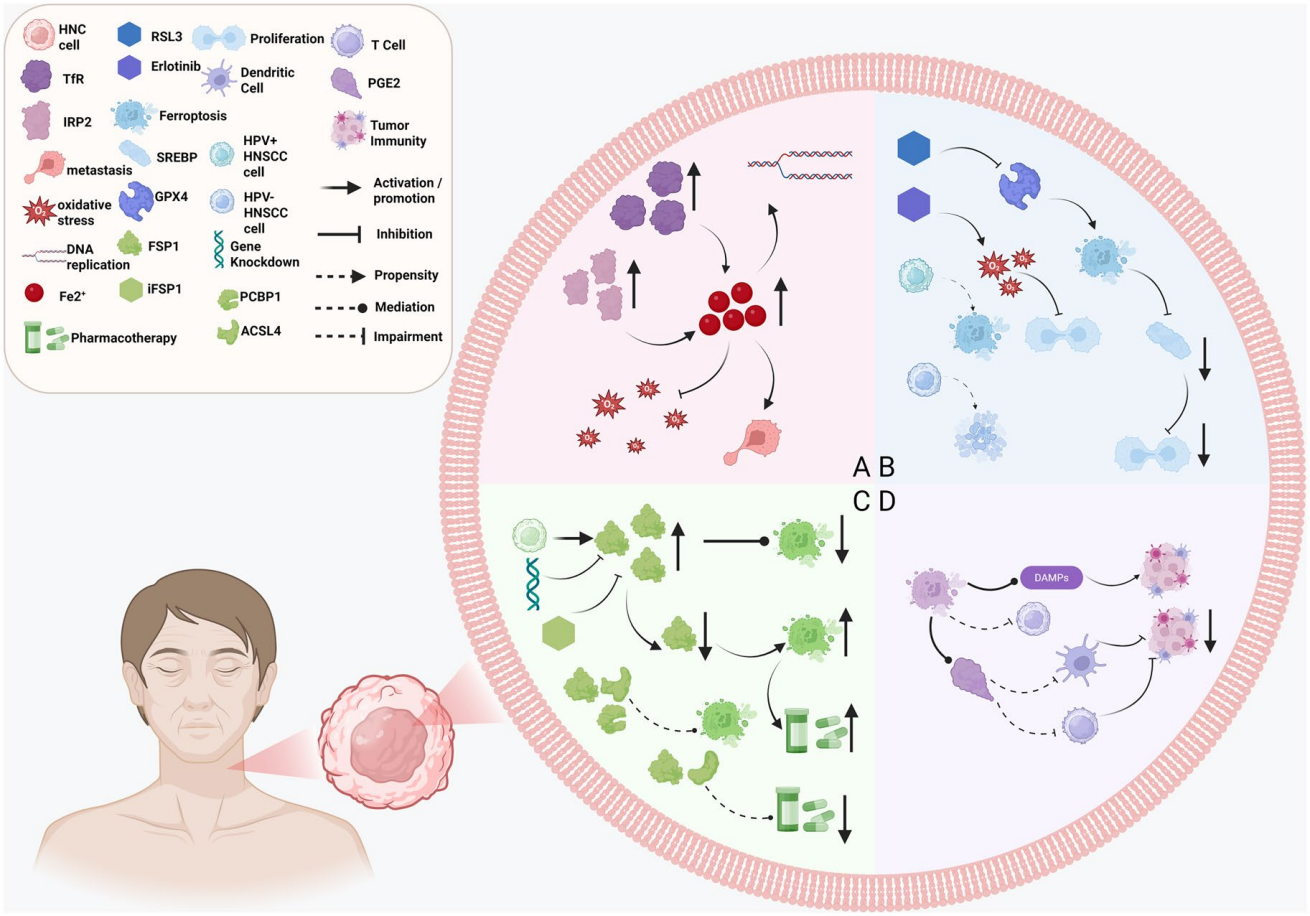
This figure illustrates the regulatory role of ferroptosis in the proliferation and survival of HNC cells: (A) HNC cells exhibit dysregulated iron metabolism and form an iron-rich microenvironment with the involvement of transferrin receptors and iron regulatory proteins. This environment supports DNA replication, enhances resistance to oxidative stress, and facilitates tumor metastasis; (B) Due to its dependence on the mesenchymal phenotype and the lipid peroxidation pathway, HNC is sensitive to GPX4 inhibition. The blockage of GPX4 can induce ferroptosis and inhibit cell proliferation by downregulating the SREBP pathway. Furthermore, HPV-positive HNSCC is more prone to ferroptosis due to its more active iron metabolism, whereas HPV-negative subtypes tend to undergo apoptosis; (C) In cisplatin-treated or recurrent HNSCC, FSP1 expression is significantly upregulated, aiding cancer cells in evading ferroptosis. Targeting FSP1 through gene knockdown or using the FSP1 inhibitor (iFSP1) can reverse drug resistance, enhance cellular sensitivity to ferroptosis, and improve chemosensitivity; (D) During early ferroptosis, cancer cells release DAMPs that activate dendritic cell-mediated anti-tumor immunity. However, ferroptosis has dual effects—it directly kills tumor cells while also releasing PGE_2_, which inhibits anti-tumor immunity by impairing dendritic cell maturation and T cell cytotoxicity.

Ferroptosis has a dual role: it can kill tumor cells but also release signaling molecules like prostaglandin E2 (PGE2), which suppresses anti-tumor immunity by impairing dendritic cell maturation and T-cell cytotoxicity [17,47]. Targeting pathways like TRIB3 or addressing PIK3CA/PTEN-related resistance highlights ferroptosis’ context-dependent effects in HNC, influencing both tumor suppression and progression through immune modulation and signaling pathway interactions [48–50] (Figure 2).

#### 2.2.2. Effects on the tumor microenvironment

**Figure 3.**
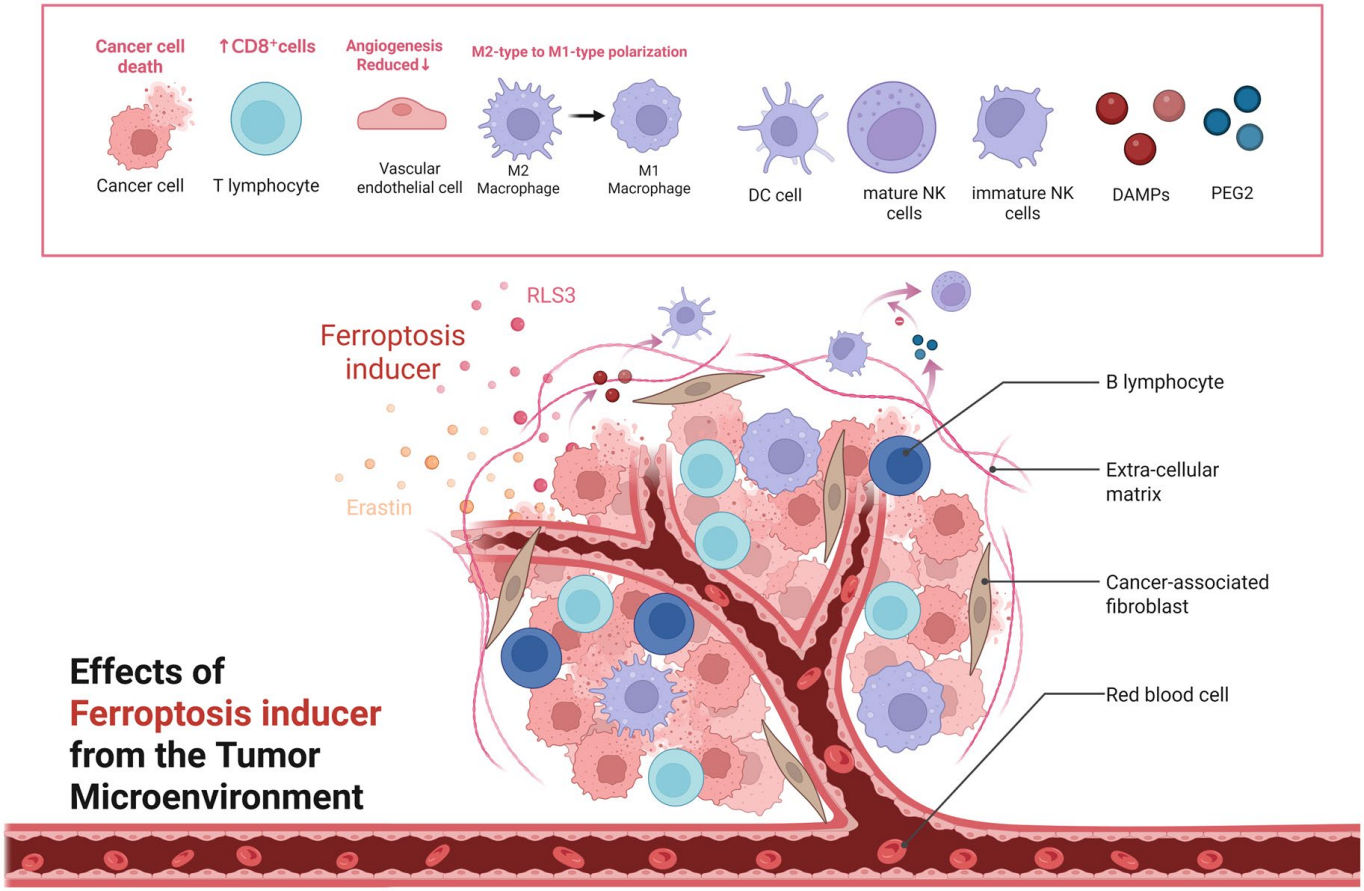
Ferroptosis inducers suppress tumor growth through direct killing of tumor cells. Within the TME, ferroptosis also exerts substantial impacts on immune regulation: it promotes the polarization of macrophages toward an anti-tumor phenotype, reduces the number and activity of immunosuppressive cells, enhances anti-tumor immune responses and immune cell functions, and regulates the expression of cytokines/chemokines, thereby synergistically potentiating the efficacy of immune checkpoint inhibitors. Furthermore, ferroptosis inducers can inhibit endothelial cell (EC) activation and suppress angiogenesis within the TME; nevertheless, they may increase vascular permeability, which potentially facilitates tumor metastasis.

Tumor cells are central to the tumor microenvironment (TME), which comprises surrounding cells and non-cellular components [51]. Ferroptosis can inhibit tumor growth by inducing tumor cell death [52]. Within the TME, ferroptosis significantly influences the polarization of macrophages. It decreases both the quantity and activity of immunosuppressive cells, boosts anti-tumor immune responses, and promotes the activation and efficacy of immune cells. Additionally, it affects the expression levels of various cytokines and chemokines, and works in conjunction with immune checkpoint inhibitors to amplify the effects of anti-tumor immunity [52–55]. Filipa et al. [56] found that ferroptosis inducers can inhibit endothelial cell (EC) activation, thereby reducing angiogenesis in the TME. However, ferroptosis inducers may also increase vascular endothelial cell permeability, promoting tumor cell metastasis [57,58] (Figure 3).

#### 2.2.3. The potential application of ferroptosis in HNC treatment

Erastin, a System Xc^-^ inhibitor, consistently induces ferroptosis in HNC cells [59,60]. Preclinical combination therapies, such as melatonin and PLK1 inhibition, effectively suppress tumor growth and elevate markers of ferroptosis without significant systemic toxicity. However, concerns regarding limited tumor selectivity and a narrow therapeutic window persist [61–63]. Translational strategies—including tumor-targeted delivery methods, such as polymeric micelles, and the development of optimized analogs—aim to maintain therapeutic efficacy while minimizing off-target effects [64].

RSL3, a direct GPX4 inhibitor, induces ferroptotic cell death in HNC cells [65]. In cases of nasopharyngeal carcinoma, RSL3 has been shown to enhance tumor ferroptosis while promoting greater infiltration of CD8^+^ T-cells [43]. Furthermore, its combination with low-dose chemotherapy significantly amplifies lipid peroxidation and cancer cell death [66]. However, the systemic inhibition of GPX4 poses a risk of injury to normal tissues. Therefore, the development of safer, tumor-selective GPX4 inhibitors or nanoparticle-based delivery systems for RSL3, in conjunction with existing therapies, may be necessary to effectively harness ferroptosis in therapy-resistant HNC.

Dihydroartemisinin (DHA), an artemisinin derivative, promotes ferroptosis largely via ROS augmentation [67]. In HNC cells, DHA induces ferroptosis and cell-cycle arrest, suppressing proliferation [68]. Tumors that are enriched in PUFA phospholipids phospholipids, facilitated by the activity of acyl-CoA synthetase long-chain family member 4 (ACSL4), exhibit increased sensitivity to this treatment [64]. Given the relatively non-specific nature of ROS and PUFA-driven ferroptosis, the therapeutic efficacy may be contingent upon the tumor’s capacity for iron handling and lipid peroxidation. Clinically, the repurposing of DHA and omega-3 PUFAs presents an attractive option; however, it will likely necessitate targeted delivery methods, such as folate-guided nanocarriers, along with biomarker-based patient selection, specifically those with high levels of ACSL4 and ALOX15.

Synergistic strategies that pair ferroptosis induction with radiotherapy and immunotherapy have shown promise in enhancing treatment efficacy. In HNC cells, statin-mediated ferroptosis has been demonstrated to sensitize radioresistant tumors to radiation [69]. Additionally, interferon-γ produced by activated T cells downregulates SLC7A11, thereby promoting ferroptosis and enhancing antitumor immunity [70]. It is crucial to carefully sequence and dose these treatments to maximize tumor ferroptosis while minimizing reactive oxygen species (ROS) injury to normal tissues and mitigating immune-related toxicity.

Ferroptosis has the potential to mitigate therapeutic resistance in HNC. For instance, erastin has been shown to overcome cisplatin resistance, while blockade of GPX4 resensitizes refractory cells to the EGFR inhibitor cetuximab [58,71]. However, tumors characterized by high levels of SLC7A11 and GPX4, or low levels of ALOX15, along with adaptive responses from NRF2 and HO-1, may remain resistant to ferroptosis. The baseline levels of SLC7A11, GPX4, and ALOX15, in conjunction with on-treatment markers of lipid peroxidation and iron, could serve as valuable indicators for patient selection and response monitoring.

Overall, ferroptosis-targeted therapy shows promise; however, it is predominantly supported by preclinical evidence [43]. Limitations of models, potential toxicities, and tumor heterogeneity hinder immediate translation, and clinical validation remains insufficient [64]. A prudent near-term approach involves repurposing well-tolerated drugs (e.g. statins, sulfasalazine) in biomarker-selected HNC cohorts to establish safety and preliminary efficacy. Future efforts should prioritize the development of novel ferroptosis agents, enhanced delivery systems, and rational combinations (e.g. dual GPX4/FSP1 inhibition), alongside rigorous safety monitoring and biomarker-guided integration with radiotherapy, chemotherapy, and immunotherapy.

## 3. The mechanism and research progress of cuproptosis in HNC

### 3.1. The discovery and molecular mechanisms of cuproptosis

#### 3.1.1. Copper ion metabolism and mitochondrial respiratory chain

**Figure 4.**
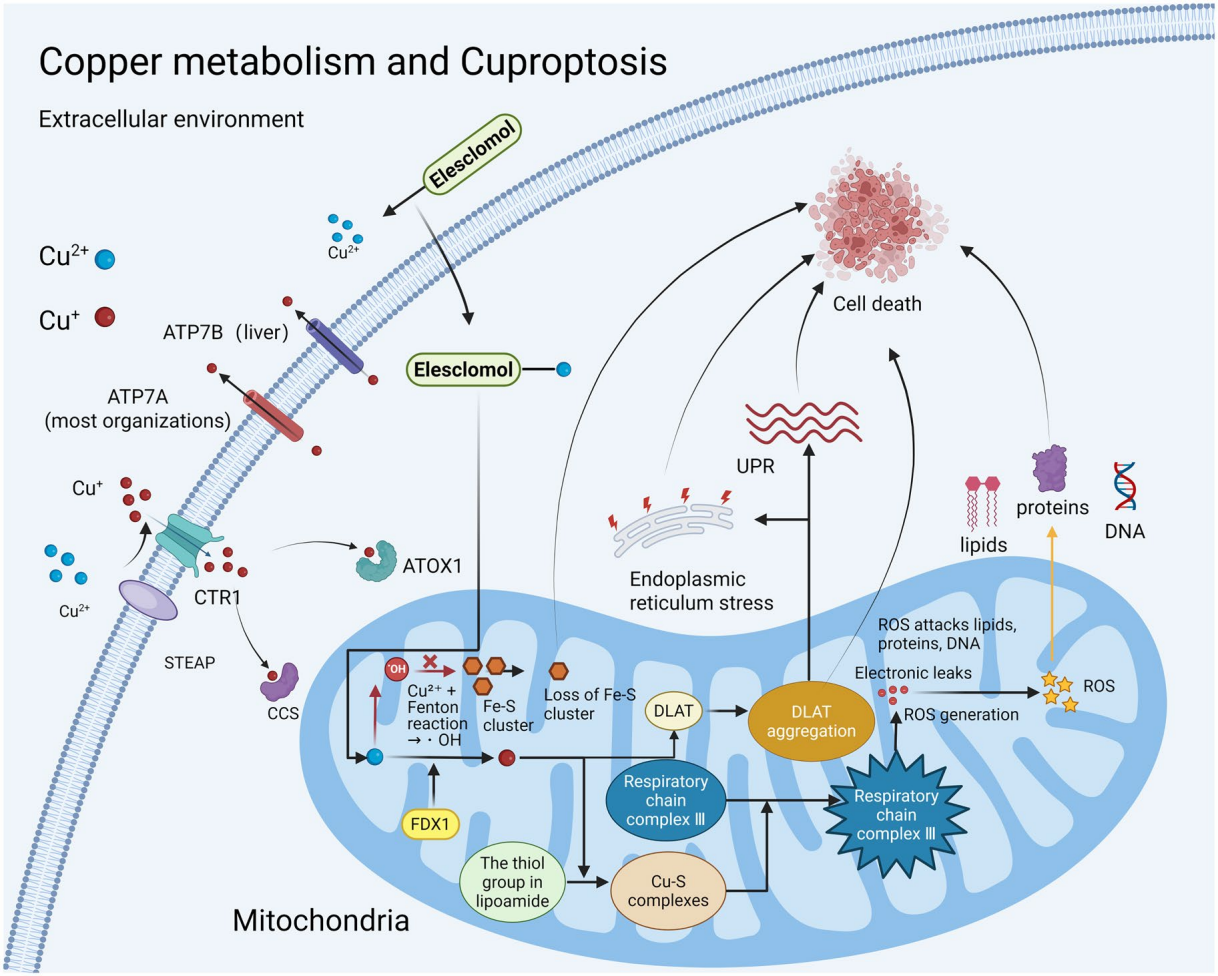
Copper ions enter cells through STEAP-mediated reduction and CTR1-transported uptake, with intracellular trafficking assisted by ATOX1 and CCS, and efflux mediated by ATP7A/ATP7B to maintain homeostasis. Excessive copper accumulation induces cuproptosis through the following mechanisms (1) Copper binds to mitochondrial lipoylated proteins, triggering their aggregation accompanied by a reduction in Fe-S cluster proteins; (2) Copper-sulfur complexes alter the conformation of respiratory chain complex III, impeding electron transport and ATP synthesis while causing electron leakage to generate ROS;(3) ROS disrupt Fe-S cluster centers via the Fenton reaction, exacerbating mitochondrial damage; (4) Copper promotes DLAT oligomerization, activating the unfolded protein response (UPR) and endoplasmic reticulum stress. These processes ultimately lead to mitochondrial membrane potential collapse, tricarboxylic acid (TCA) cycle dysfunction, and irreversible cell death.

Copper is a vital trace element in the human body, essential for various biological functions, including energy metabolism. It serves as a key cofactor for numerous enzymes, playing a crucial role in intracellular redox reactions [72,73]. Copper ions are reduced by STEAP, transported into cells by CTR1, and assisted by ATOX1 and CCS during transport. ATP7A and ATP7B are responsible for copper export, maintaining intracellular copper balance, and both play key roles in copper metabolism in most tissues and the liver, respectively [74,75]. In a groundbreaking study conducted by Tsvetkov and his team in 2022, a newly identified phenomenon known as cuproptosis was unveiled; this refers to an unique form of cell death induced by the buildup of intracellular copper. A key feature of cuproptosis is the aggregation of lipoylated mitochondrial proteins that connect with copper, which aligns with a reduction in iron-sulfur (Fe-S) cluster proteins. This interaction causes proteotoxic stress, ultimately culminating in cell death [76,77]. Mitochondria are the primary site of cuproptosis, where oxidative damage occurs at the mitochondrial membrane, impairing the function of TCA cycle enzymes during this process [78]. The mitochondrial respiratory chain consists of five complexes, with complex III being crucial in the electron transport chain, responsible for electron transfer and maintaining mitochondrial membrane potential [79]. Copper interacts with lipoamide, resulting in the formation of a Cu–S complex. This interaction modifies the conformation of complex III, disrupts electron transfer, influences ATP production, and causes electron leakage, ultimately leading to the generation of reactive oxygen species (ROS) [80]. The accumulation of ROS damages intracellular substances, while copper ions also directly attack iron-sulfur centers through the Fenton reaction, causing degradation of iron-sulfur cluster proteins and exacerbating mitochondrial dysfunction [73]. Additionally, copper promotes the oligomerization of DLAT, stimulates the unfolded protein response (UPR) along with endoplasmic reticulum stress, and the depletion of Fe-S cluster proteins results in the failure of the mitochondrial membrane potential, ultimately initiating cell death [77] (Figure 4).

#### 3.1.2. Related genes and signaling pathway regulation

The genes associated with the regulation of cuproptosis include ferredoxin 1 (FDX1), lipoic acid synthetase (LIAS), DLAT, pyruvate dehydrogenase E1 subunit (PDHB), and glycine cleavage system H protein (GCSH). The enzymes encoded by these genes form stable polymers through lipoylation modification, disrupting mitochondrial protein homeostasis [77] (Table 1).

**Table 1.** Genes and signaling pathways involved in cuproptosis in HNC.

Genes/Signaling pathways	Functional description	Mechanism of action in copper death	Association with head and neck cancer	Findings and conclusions	References
FDX1	Intracellular participation in electron transport processes and maintenance of intracellular REDOX balance	1. The reduction of Cu^²+^ to stronger toxic Cu^+^ promotes Cu^+^ overload within the mitochondria 2. regulation of lipoacylated protein stability, leading to mitochondrial toxic stress 3. Elevated activity promotes the accumulation of copper in mitochondria, induces the lipid acylation modification of TCA cycling-related enzymes (such as DLAT, DLST), and leads to toxic protein aggregation and mitochondrial dysfunction	HNSCC is negatively correlated with malignant progression of tumor and T cell depletion	FDX1 knockdown completely resists cell death induced by copper ions 2. It is suggested that it acts as a key molecular switch of copper death	[73]
LIAS	Responsible for lipoic acid synthesis and maintaining normal mitochondrial lipid acylation protein function	1. The synthesized lipoic acid is a cofactor of DLAT, which can bind to copper and trigger copper death 2. Excess Cu^+^ suppresses its activity and prevents normal lipoic acid synthesis, further aggravating copper death	Low expression in OSCC indicates suppression of copper death	Enhanced LIAS expression can promote cell resistance to copper death	[81–85]
PDHB	The main subunit of pyruvate dehydrogenase (PDH). PDH catalyzes the decarboxylation of pyruvate and participates in the glycolytic pathway, thereby regulating cellular energy metabolism.	It may indirectly influence the generation of reactive oxygen species (ROS) by modulating the activity of the tricarboxylic acid (TCA) cycle. Excessive accumulation of copper can disrupt its function, resulting in metabolic disorders that exacerbate ROS production.	Low expression in OSCC indicates that cuproptosis is suppressed.	Enhancing PDHB expression can promote cellular cuproptosis.	[85–88]
GCSH	The key component of the Glycine Cleavage System (GCS), responsible for catalyzing the cleavage reaction of glycine, and involved in the synthesis of GSH.	By synthesizing the antioxidant GSH, it counteracts the effects of ROS and inhibits cuproptosis.	Highly expressed in HNC and associated with poor prognosis in tumors.	Knockdown of GCSH inhibits tumor cell proliferation, migration, and invasion. 2. It can serve as a prognostic indicator related to cuproptosis in cancer.	[89,90]
KEAP1- NRF2	KEAP1 is a negative regulator of NRF2, which plays an important role in how cells respond to oxidative stress, inflammation, and cell damage	1. Under normal physiological conditions, KEAP1 degrades NRF2 through ubiquitination, limiting the activation of ARE, and reducing the resistance of cells to copper death 2. When the cells are exposed to oxidative stress such as copper overload, the conformational change of KEAP1 leads to the release and incorporation of NRF2 into the nucleus, the activation of glutathione (GSH) synthesis, thioredoxin (TXN) and other antioxidant genes, and the alleviation of cup-induced mitochondrial oxidative damage	About 15%-30% of HNSCC patients have KEAP1 dysfunction mutations, resulting in sustained NRF2 pathway activation. In HNSCC, high NRF2 activity promotes tumor resistance by enhancing antioxidant defense and reducing cupr-induced cell death.	1. It is suggested that restoring KEAP1 function or inhibiting NRF2 function is a feasible way to reverse copper death resistance in HNSCC patients	[8,48, 78,91,92]

Notably, the KEAP1-NRF2 signaling pathway plays a complex role in regulating not just cuproptosis, but also ferroptosis and apoptosis, despite these processes being controlled by different regulatory mechanisms. Ferroptosis, a specific form of regulated cell death, primarily depends on lipid peroxidation associated with iron and is closely connected to decreased levels of glutathione (GSH) and the inactivation of GPX4 [9]. NRF2 inhibits lipid peroxidation and antagonizes ferroptosis by upregulating glutathione synthase (GCLC/GCLM) and thioredoxin (TXN) [93]. There exists a synergistic and antagonistic relationship betwn cuproptosis and ferroptosis. Cuproptosis inhibits the synthesis of iron-sulfur cluster proteins, releasing Fe^2+^ to promote ferroptosis[94]. A study conducted by Wang and colleagues [95] showed that inducers of ferroptosis, including sorafenib and erastin, markedly increased the cell death triggered by copper ionophores in primary liver cancer cells. When using a combination of these ferroptosis inducers with the cuproptosis inducer Elesclomol (ES), researchers observed a notable increase in the aggregation of lipoylated DLAT, which suggests that this combination effectively promotes cuproptosis. Thus, it can be concluded that copper significantly contributes to the induction of proteotoxic stress, which not only leads to cuproptosis but also influences ferroptosis by modulating the degradation of GPX4 [96].

This series of complex interactions collectively constructs a delicate network of cell death regulation, providing important clues for a deeper understanding of the mechanisms of tumorigenesis and development, as well as for the development of novel therapeutic strategies.

### 3.2. The relationship between cuproptosis and HNC

#### 3.2.1. Impact on the biological behavior of HNC cells

Cuproptosis has a variety of impacts on the biological characteristics of head and neck cancer cells, as outlined below. It particularly negatively influences the growth of cells with hyperactive copper/iron metabolism, such as HPV-positive HNC subtypes. Shimada et al. [97] indicates that dysregulation of copper ion metabolism leads to DNA damage, subsequently causing cell cycle arrest. This process not only restrains the proliferation of cells but also triggers apoptosis [98]. According to Li et al. [81], both LIAS and PDHB are downregulated in cancerous tissues compared to adjacent oral squamous cell carcinoma (OSCC) tissues, whereas GLS and CDKN2A are upregulated. This indicates that the cuproptosis mechanism in tumor cells is hindered, which in turn supports tumor growth. Additionally, cuproptosis can facilitate metastasis and invasion of cancer cells. In the Notch signaling pathway, the presence of copper facilitates the release of the Jagged1 ligand, thereby boosting the mobility of cancer cells [99]. The level of copper ions in the tumor tissues of HNSCC patients is significantly higher than in normal tissues and is positively correlated with TNM staging. Patients in stages III–IV exhibit higher copper ion levels than those in stages I–II, suggesting that cuproptosis may promote invasive tumor growth through oxidative stress and DNA damage [100]. Elevated copper ion levels in tumor tissues correlate with tumor stage, LNM, and poor prognosis. Copper-induced cell death promotes tumor angiogenesis and immune evasion by activating pathways such as HIF-1α and NF-κB [101]. Research by Wang et al. [100] indicates that FDX1 is a key gene involved in copper-induced cell death, showing a significant negative correlation with malignant progression and T cell exhaustion in HNSCC. Literature suggests that elevated copper levels are positively correlated with LNM [102]. In other words, copper levels may serve as an important biomarker for the early assessment of the risk of LNM.

#### 3.2.2. Association with HNC treatment

Cuproptosis, is promising for HNC due to cancer cells’ high copper metabolism [103]. Cuproptosis-inducing drugs, mainly copper ionophores (e.g. Elesclomol, Disulfiram), and copper chelators act via enhancing or reducing intracellular copper levels, respectively [104].

Elesclomol forms a membrane-permeable Cu^2+^ complex to transport copper to mitochondria, while Disulfiram creates CuET to facilitate copper uptake [103–106]. Combinatorial therapies show synergy: NP@ESCu paired with αPD-L1 reduces tumor growth in preclinical HNSCC models by increasing PD-L1 expression and anti-tumor immunity, and Disulfiram overcomes resistance to multiple chemotherapeutics via ROS modulation [104,107]. Targeting NRF2 resistance or combining with anti-angiogenic agents (e.g. Bevacizumab) further enhances efficacy [108].

Yang et al. [109] found that hypoxia-inducible factor-1α (HIF-1α) is a key driver of copper-related chemotherapy resistance in solid tumors. They discovered that HIF-1α activates pyruvate dehydrogenase kinase 1 and PDK1/3, leading to reduced expression of dihydrolipoamide S-acetyltransferase (DLAT), a copper target, and promoting the accumulation of metallothioneins. Metallothioneins chelate mitochondrial copper, resulting in resistance to copper toxicity under hypoxic conditions

Li et al. [110] demonstrated that ORL@Cu-MOF effectively triggers cuproptosis and suppresses fatty acid metabolism, exhibiting anti-tumor effects and remodeling the TME in mouse models. The combination of ORL@Cu-MOF with programmed cell death receptor-1 (αPD-1) significantly enhances immunotherapy efficacy, converting ‘cold tumors’ into ‘hot tumors’.

Copper-based nanomaterials like NP@ESCu and CuD@PM enable precise, ROS-responsive copper delivery, improving bioavailability and reprogramming the immunosuppressive microenvironment (e.g. promoting CD8^+^ T cell infiltration) [107,111]. Despite preclinical promise, clinical translation faces hurdles: short drug half-life, poor tumor penetration, and heterogeneous patient populations. Leveraging biomarkers, gene editing, and imaging for patient stratification, alongside developing targeted nanoparticles, could address these challenges. Ethical concerns about off-target toxicity and long-term copper imbalance require stratified informed consent and safety monitoring, highlighting the need for robust clinical trial design to validate cuproptosis-based therapies.

## 4. The mechanism and research progress of disulfidptosis in HNC

### 4.1. Mechanism of disulfidptosis

#### 4.1.1. Disulfide stress and protein homeostasis

Disulfidptosis represents a newly identified modality of cell death, as proposed by Liu et al. in 2023. This distinctive process is marked by an accumulation of excessive intracellular disulfides, which culminates in disulfide stress and a subsequent failure of the actin cytoskeleton [112]. Notably, disulfidptosis predominantly affects tumor cells exhibiting elevated levels of SLC7A11. This condition is particularly triggered in environments characterized by glucose starvation or a deficiency in cysteine [112,113]. The role of SLC7A11 is crucial, as it mediates the transport of cystine and glutamate, essential metabolites influencing cellular functions. When glucose is abundant, cells rely on the pentose phosphate pathway (PPP) to generate NADPH, maintaining redox balance. However, when glucose supply is limited, the PPP is inhibited, resulting in decreased NADPH levels and ineffective reduction of cystine. The continuous uptake of cystine by SLC7A11 exacerbates NADPH depletion, triggering disulfide stress that ultimately leads to cytoskeletal collapse and cell death [112].

Protein disulfide isomerase (PDI) plays a pivotal role within the endoplasmic reticulum, where it is tasked with facilitating proper protein folding and sustaining the balance of redox homeostasis [114,115]. When NADPH is depleted, the accumulation of cystine and cysteine leads to the oxidation of protein thiols into disulfide bonds, disrupting protein conformation and function, which results in cytoskeletal disintegration. Excessive formation of disulfide bonds can also trigger endoplasmic reticulum stress (ERS) [114,116].

ERS is sensed by three transmembrane proteins—PERK, IRE1α, and ATF6—that together reestablish proteostasis. When misfolded or disulfide‑crosslinked proteins accumulate, BiP (GRP78) dissociates, activating each UPR branch. The PERK–eIF2α–ATF4 axis limits new protein synthesis and upregulates antioxidant and thiol‑reducing enzymes to control aberrant disulfide bonds. Likewise, IRE1α/XBP1 signaling induces folding catalysts and ERAD components, while ATF6 activation requires reduction of its own disulfide bonds by ER oxidoreductases (e.g. ERp18/PDIA5) before Golgi cleavage [115]. Together, these pathways boost both correct disulfide formation and disulfide‑bond resolution. In disulfidptosis—cell death driven by overwhelming disulfide stress—these UPR responses act protectively: intact UPR delays disulfidptosis, whereas inhibiting PERK or IRE1α markedly sensitizes SLC7A11‑high cells to glucose‑starvation‑induced disulfidptosis [117]. Under such conditions, unchecked accumulation of disulfide‑crosslinked proteins leads to cytoskeletal collapse. Conversely, mild ER stress induction can rescue cells from disulfidptosis. This supports combining glucose‑transporter inhibitors with UPR pathway inhibitors to promote disulfidptosis in cancer therapy.

#### 4.1.2. Metabolic pathways, enzymes, and gene regulation

In disulfidptosis, the glutaredoxin (Grx) and thioredoxin (Trx) antioxidant systems, along with protein disulfide isomerase (PDI), participate in the formation of disulfide bonds, regulate disulfide stress, and maintain intracellular redox homeostasis [116]. The Grx system, composed of Grx, glutathione (GSH), glutathione reductase (GR), and nicotinamide adenine dinucleotide phosphate (NADPH), is involved in glutathionylation, modulating protein activity and cell signaling while protecting against oxidative damage [118–120]. The Trx system, consisting of Trx, thioredoxin reductase (TrxR), and NADPH [121], works in concert to alleviate oxidative stress. Collectively, these systems enhance cellular defense and maintain redox homeostasis [116]. PDI is involved in endoplasmic reticulum protein folding, while endoplasmic reticulum oxidase 1 (ERO1) interacts with it to regulate intracellular reactive oxygen species (ROS) levels [114].

**Figure 5.**
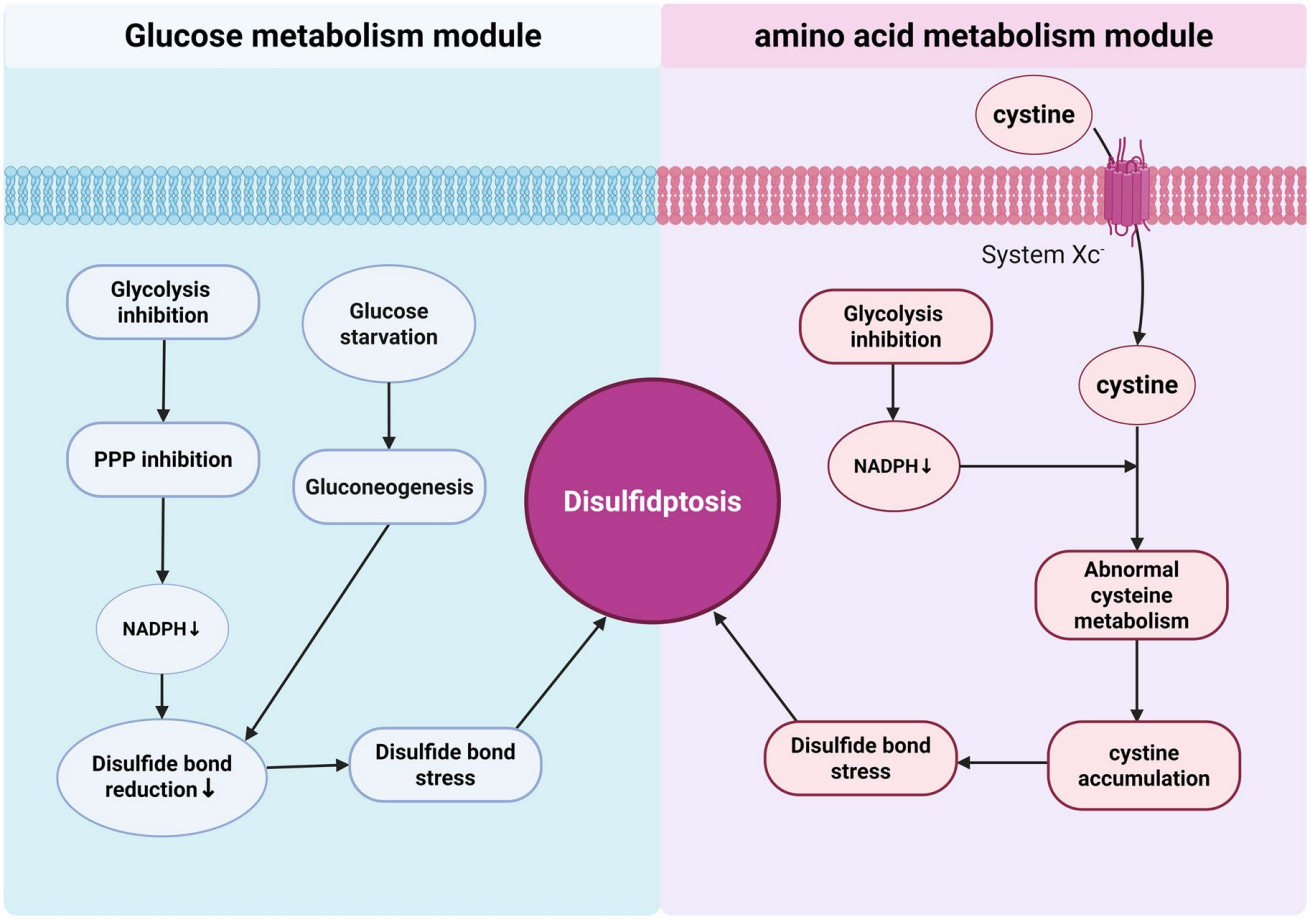
This figure illustrates the mechanisms by which glucose and amino acid metabolism modules regulate disulfidptosis. The left panel presents the glucose metabolism module, wherein the inhibition of glycolysis or glucose starvation modulates pathways such as the PPP and gluconeogenesis, ultimately leading to a decrease in NADPH levels. This reduction impairs the reducing capacity of disulfide bonds, induces disulfide stress, and consequently promotes disulfidptosis. Conversely, the right panel depicts the amino acid metabolism module, where cystine is transported into cells *via* System Xc^-^ and subsequently converted to cysteine. The depletion of NADPH, resulting from glycolysis inhibition, disrupts cysteine metabolism, leading to cystine accumulation. This accumulation triggers disulfide stress, which further contributes to the regulation of disulfidptosis. Collectively, these metabolic modules regulate the process of disulfidptosis through the interplay of NADPH homeostasis, disulfide bond dynamics, and cysteine metabolism.

Disulfidptosis interacts with core metabolic pathways such as glucose metabolism and amino acid metabolism. During glucose starvation, the decrease in NADPH produced by the PPP reduces the capacity for disulfide bond reduction, triggering disulfidptosis. Inhibition of the glycolysis pathway also affects sensitivity to this form of cell death [112,122]. At this stage, PPP is obstructed, NADPH is depleted, cysteine metabolism becomes abnormal, cystine accumulation triggers disulfide stress, and gluconeogenesis of glucogenic amino acids exacerbates the imbalance, all of which can induce disulfide cell death [123–125] (Figure 5).

The Rac1-WAVE regulatory complex signaling pathway (Rac1-WRC) significantly influences disulfide cell death [126]. As a core member of the Rho protein family, Rac1, together with the WAVE regulatory complex (WRC), mediates branched actin polymerization, increasing the sensitivity of the actin cytoskeleton to disulfide stress and promoting disulfide cell death. The absence of components such as NCKAP1 in the WRC inhibits disulfide cell death [127].

The induction of disulfidptosis is governed by several key genes and signaling pathways. At the metabolic level, SLC7A11 is the core effector: its high expression endows cells with resistance to oxidative stress, but also creates a vulnerability to disulfide stress under glucose deprivation. The role of SLC7A11 is context-dependent – moderate overexpression is beneficial for cell survival, whereas excessively high expression leads to cell death under oxidative pressure [128]. Upstream, transcription factors such as NRF2 (NFE2L2) and ATF4 are known to upregulate SLC7A11 transcription, conversely, the tumor suppressor BAP1 represses SLC7A11 expression *via* H2A deubiquitination, thereby modulating cellular susceptibility to disulfidptosis [129]. Wang et al. [129] demonstrated that high BAP1 expression protects cells from glucose-starvation-induced disulfidptosis, and this protection is abrogated by SLC7A11 overexpression or excess cystine supplementation. This implies that tumors with BAP1 loss-of-function (e.g. certain renal cancers) lack SLC7A11 restraint and become more prone to disulfidptosis under stress conditions.

### 4.2. The association between disulfidptosis and HNC

#### 4.2.1. Impact of disulfidptosis on the survival and metabolism of HNC cells

HNC cells, as tumor cells, predominantly depend on the glycolytic pathway for energy metabolism. The occurrence of disulfidptosis in these cells results in the depletion of NADPH, which adversely affects the activity of several key enzymes in the glycolytic pathway, subsequently influencing the production of glycolytic products and overall cellular energy metabolism [127,130]. Furthermore, NADPH depletion exacerbates oxidative stress, leading to metabolic collapse [131]. Compared to normal cells, HNC cells exhibit elevated expression levels of SLC7A11, rendering them more vulnerable to disulfidptosis [132]. During glucose starvation, the activity of critical glycolytic enzymes, such as PKM2, is inhibited, resulting in decreased ATP production and simultaneously limiting the NADPH supply from the pentose phosphate pathway (PPP), which leads to an insufficient reducing capacity [132]. Disulfidptosis primarily transpires in the cytoplasm, particularly within the cytoplasmic matrix and the actin cytoskeleton. Additionally, disulfide stress also impacts the endoplasmic reticulum, triggering endoplasmic reticulum stress that further exacerbates cellular damage and death [112]. Currently, research on disulfidptosis in HNC cells is relatively sparse, predominantly focusing on genes associated with disulfidptosis and the predictive role of long non-coding RNAs in tumor prognosis [133,134]. Based on relevant bioinformatics analyses, it can be inferred that disulfidptosis is closely linked to the growth and survival of HNC cells.

#### 4.2.2. Potential applications of disulfidptosis in HNC treatment

In the realm of cancer therapy, disulfidptosis, akin to other metabolic forms of cell death such as ferroptosis, holds significant potential for therapeutic interventions. The crucial role of SLC7A11 in disulfidptosis provides a pathway for the development of drugs specifically aimed at inducing disulfidptosis. Studies have demonstrated that glucose transporter inhibitors (e.g. BAY-876) can induce disulfidptosis by obstructing glucose uptake, which leads to NADPH depletion in tumor cells with elevated SLC7A11 expression [112]. The integration of disulfidptosis with existing therapeutic modalities can substantially enhance therapeutic efficacy. Wang et al. [113] found that the combined application of GLUT inhibitors and endoplasmic reticulum stress inhibitors significantly augmented the disulfidptosis effect and inhibited tumor cell proliferation. Moreover, disulfidptosis can release tumor antigens and activate immune responses. Zhu et al. [135] demonstrated that the combination of nanodrugs and PD-1 inhibitors might enhance anti-tumor immunity through a ‘dual death mode’ (pyroptosis + disulfidptosis). Research has indicated that SLC7A11 is highly expressed in HNC, making these cells more predisposed to disulfidptosis [136]. Due to the heightened sensitivity of HNC cells to disulfidptosis, the utilization of agents that induce disulfidptosis, along with the upregulation of related genes like SLC7A11, could enhance the vulnerability of these cancer cells to this form of cell death. This approach not only presents innovative therapeutic strategies but also identifies novel targets for effective treatment.

Despite these promising prospects, the application of disulfidptosis faces several challenges. First, the high heterogeneity of SLC7A11 expression in HNC may affect therapeutic efficacy, necessitating the use of single-cell sequencing or metabolic imaging technologies to identify sensitive subpopulations. Second, normal tissues, such as the liver and kidneys, also rely on SLC7A11, highlighting the need for targeted delivery systems (e.g. antibody-drug conjugates or nanocarriers) to mitigate systemic toxicity. Finally, tumor cells may compensate for NADPH deficiency through glutamine metabolism or fatty acid oxidation, necessitating the combination of metabolic pathway inhibitors (e.g. the glutaminase inhibitor CB-839) to block potential escape routes. Disulfidptosis offers a novel direction for the precise treatment of HNC. As mechanistic research deepens and combination strategies are optimized, disulfidptosis is expected to become a vital component of the comprehensive treatment system for HNC, providing breakthrough therapeutic options, particularly for drug-resistant or advanced-stage patients.

## 5. Prospects of lysozincrosis and alkaliptosis in HNC

### 5.1. Overview of lysozincrosis

Lysozincrosis was first proposed by Du et al. in 2021. This process involves the activation of the lysosomal calcium channel transient receptor potential mucolipin 1 (TRPML1) by the TRPML1 agonist ML-Sas, leading to the release of excessive Zn2+ from lysosomes. This release results in mitochondrial damage and rapid ATP depletion, ultimately triggering lysozincrosis [137]. In HNC, lysosomal function is often upregulated due to the high metabolic demands of tumor cells. The abnormally high expression of TRPML1 in metastatic tumors may serve as a specific target for therapy [137–139]. Research on cell death induced by lysosomal zinc ionophores (LCD) in HNC is currently limited. In the future, activating TRPML1 to induce lysosomal zinc ionophore-induced cell death may become a significant strategy for treating HNC.

### 5.2. Overview of alkaliptosis

Alkaliptosis represents a newly identified type of cellular demise prompted by the specific inhibitor JTC801, which leads to an increase in intracellular alkalinity through the blockage of the opioid receptor-like 1 (OPRL1). This process of alkaliptosis is significantly linked to the functioning of the NF-κB signaling pathway, and the suppression of NF-κB effectively diminishes intracellular alkaliptosis. Carbonic Anhydrase 9 (CA9) serves as a crucial regulator of intracellular pH and plays a significant role in this process by managing the reversible conversion of carbon dioxide into bicarbonate. The activation of NF-κB leads to the inhibition of CA9, which in turn facilitates alkaliptosis. JTC801 selectively triggers alkaliptosis in cancerous cells by taking advantage of the varying expression levels of pH regulation found in normal versus cancer cells. Additionally, established techniques for preventing cell death are ineffective in halting alkaliptosis, highlighting its distinct nature [140,141].

### 5.3. Limitations of current research

There is currently a lack of direct evidence on alkaliptosis and disulfidptosis in HNC. Most conclusions are based on extrapolations from other cancers, and specific models for HNC are urgently needed for validation. While some studies have explored these forms of cell death in other cancers, their mechanisms and clinical relevance in HNC remain unclear. Consequently, the potential of alkaliptosis and disulfidptosis in this context is speculative, requiring more data to confirm their roles.

Additionally, the clinical application of these mechanisms is uncertain, as inducers like Elesclomol and JTC801 have not yet entered clinical trials for HNC, and their safety and efficacy are still unproven. To address these gaps, future research should focus on developing HNC-specific models and conducting preclinical studies to assess the safety and efficacy of these inducers. Exploring combination therapies with existing treatments could also provide new therapeutic options.

### 5.4. Perspectives on lysozincrosis and alkaliptosis in HNC

Currently, there is insufficient literature examining lysozincrosis and alkaliptosis in relation to HNC. However, based on research concerning these two mechanisms of cell death in various cancer types, it is plausible to anticipate their possible contributions to the treatment of HNC. TRPML1 serves as a crucial target for lysozincrosis [137–139]. Given the enhanced lysosomal function in HNC, inducing lysozincrosis with TRPML1 agonists may serve as an effective supplement to existing treatment strategies for HNC. Considering the toxic effects of zinc ions on normal cells, developing nanocarriers for localized enrichment of zinc ions in tumors could effectively reduce systemic toxicity. Additionally, the advancement of genes related to TRPML1 and zinc homeostasis shows potential as future biomarkers for predicting the prognosis of HNC. Alkaliptosis, characterized by its selective killing of cancer cells, offers a safe and effective strategy for the treatment of refractory cancers such as HNC [140]. Based on the characteristics of alkaliptosis, utilizing alkaliptosis inducers (such as CA inhibitors and sodium-hydrogen exchanger (NHE) inhibitors) can selectively induce alkaliptosis in HNC cells, thereby reducing the toxic side effects of drugs [141]. Further research into the mechanisms of lysozincrosis and alkaliptosis will help elucidate the interactions between lysozincrosis and autophagy and apoptosis, as well as the cross-regulatory mechanisms between the NLRP3 inflammasome and other death pathways in alkaliptosis. Future studies may employ single-cell sequencing alongside spatial metabolomics techniques to unravel the zinc metabolism network present in the microenvironment of HNC. Concurrently, preliminary clinical trials of TRPML1 agonists or CA inhibitors should be initiated as soon as possible to assess their safety and efficacy.

## 6. Interaction and network regulation of multiple metabolic cell death pathways in HNC

**Figure 6.**
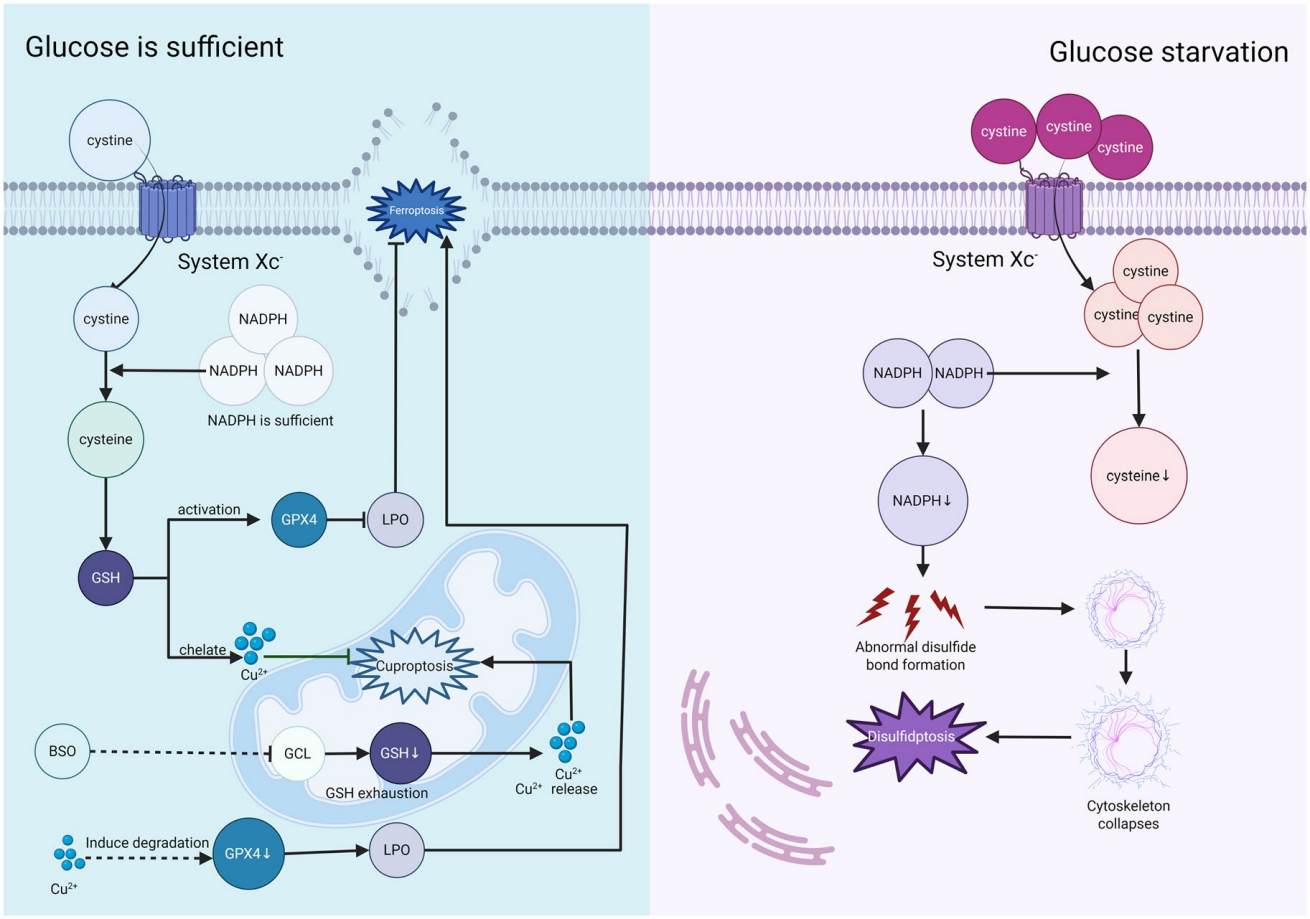
SLC7A11 bidirectionally regulates cell death through cystine transport: under glucose-sufficient conditions, its high expression maintains GSH synthesis to inhibit ferroptosis; under glucose-deficient conditions, it mediates cystine overload, leading to NADPH depletion and triggering disulfidptosis. High expression of SLC7A11 in OSCC predicts lymphatic/nerve invasion and poor prognosis. GSH acts as a hub molecule: it not only serves as a cofactor of GPX4 to resist ferroptosis, but also chelates copper to inhibit cuproptosis. Depletion of GSH by BSO can release copper chelation and promote cuproptosis. Copper ions synergistically promote ferroptosis by inducing autophagic degradation of GPX4, forming an interactive network of cell death modes.

Ferroptosis and disulfidptosis are related to SLC7A11 - mediated cystine transport. In the tissues of patients with OSCC, the expression of SLC7A11 is significantly associated with lymphatic invasion and perineural invasion, serving as an independent predictor of poor prognosis [142]. In HNC cells, high SLC7A11 expression inhibits ferroptosis by maintaining GSH synthesis when glucose is sufficient, but cystine over - accumulation depletes NADPH, causing disulfide stress and cell death [9,112]. Glucose availability decides SLC7A11’s role: it promotes disulfidptosis without glucose and inhibits ferroptosis with plenty of glucose [143]. GSH is a key GPX4 cofactor against ferroptosis and a copper - ion chelator to inhibit cuproptosis. BSO - induced GSH depletion promotes cuproptosis [76]. Moreover, copper can trigger GPX4 autophagic degradation, facilitating ferroptosis [96,143](Figure 6).

In metastatic lesions of HNC, disulfidptosis may demonstrate enhanced therapeutic efficacy. Clinical samples indicate that SLC7A11 levels are higher in HNSCC metastases compared to primary lesions and are associated with poorer prognosis, suggesting that primary lesions (which exhibit lower SLC7A11 levels) are more responsive to ferroptosis therapy, while metastatic lesions may exhibit greater resistance [43,144]. In HNSCC cell lines and mouse models, the inhibition of System Xc^-^ or direct targeting of GPX4 induces significant ferroptosis and suppresses tumor growth; however, the therapeutic effect is diminished in strains with high SLC7A11 expression, indicating the necessity for patient stratification based on SLC7A11 expression levels [58]. Given that SLC7A11 is upregulated in metastatic HNSCC compared to primary tumors, and that disulfidptosis selectively targets cells with high SLC7A11 expression, it can be theorized that disulfidptosis holds a specific advantage over ferroptosis in selectively targeting metastatic or recurrent lesions—particularly in the context of targeted therapy for such lesions [112]. Although bioinformatics-based studies have indicated the predictive value of disulfidptosis-related genes in HNSCC prognostic models, *in vivo* and *in vitro* validation specifically for metastatic lesions remains lacking, necessitating further preclinical research [145,146].

Currently, there is no evidence or research indicating direct interactions or common regulatory factors between lysozincrosis, alkaliptosis, and the aforementioned three types of cell death. Current research primarily focuses on other types of cell death mechanisms, with a lack of validation in specific models of HNC. As a result, the precise mechanisms of lysosomal zinc-mediated cell death and alkalosis-induced cell death in HNC remain unclear. Further experimental and clinical data are urgently needed to confirm their roles in tumor biology.

In summary, HNCs exhibit complex interactions and network regulation among metabolic cell death pathways, including ferroptosis, cuproptosis, disulfidptosis, lysozincrosis, and alkaliptosis. Future research should prioritize elucidating the direct mechanisms of interaction among these metabolic cell death pathways, particularly the relationships between lysosomal zinc-dependent cell death and alkaliptosis with other pathways. This will help in deciphering the complex regulatory systems that control the survival and death of cancer cells in the head and neck region, laying a theoretical basis for formulating novel therapeutic approaches. Additionally, comprehensive investigation into the mechanisms of these metabolic pathways leading to cell death in HNC may reveal new targets for treating resistant cases, ultimately boosting treatment effectiveness and improving patient outcomes.

## 7. Synergistic effects of metabolic cell death mechanisms with conventional treatments in HNC

Metabolic cell death modalities, such as ferroptosis and cuproptosis, exhibit synergistic and mutually reinforcing effects when combined with traditional cancer treatments. Radiotherapy generates a substantial amount of reactive oxygen species (ROS) and can upregulate ACSL4, which promotes the accumulation of polyunsaturated fatty acid phospholipids (PUFA PL) in cell membranes, thereby enhancing lipid peroxidation and activating the ferroptosis pathway, significantly increasing the lethality of radiotherapy [147]. Furthermore, ferroptosis inducers, including Erastin and Sulfasalazine, can substantially reverse radiotherapy resistance and improve radiotherapy efficacy by inhibiting System Xc^-^ or GPX4, which blocks glutathione (GSH) synthesis and increases ROS and lipid peroxidation levels [54,58,69,148]. F.V et al. [149] demonstrated that the ROS inducer MitoTam can induce ferroptosis in HNSCC cells and significantly inhibit the survival of radiotherapy-tolerant cells during subsequent radiotherapy, providing a novel strategy for targeting radiotherapy-resistant tumors. Research indicates that in HNC, the combination of chemotherapeutic agents, such as cisplatin, with ferroptosis inducers can synergistically enhance tumor cell apoptosis and ferroptosis by increasing lipid peroxidation and reducing GSH levels, thereby improving the efficacy of chemotherapy [58].

Radiation therapy can lead to mitochondrial copper accumulation and lipoyl protein aggregation by upregulating CTR1 (copper transporter 1) and depleting mitochondrial glutathione, thereby triggering cuproptosis [150].

Ferroptosis has shown tremendous potential in tumor immunotherapy. Studies have demonstrated that ferroptosis can enhance the immunogenicity of tumor cells and improve the efficacy of immunotherapy through interactions with immune cells [151]. For example, ferroptosis inducers can enhance the efficacy of immune checkpoint inhibitors (such as PD-1/PD-L1 antibodies) by regulating the tumor immune microenvironment [152]. Moreover, the synergistic effect between ferroptosis and immunotherapy has been validated in various tumor models, including triple-negative breast cancer, colon cancer, and pancreatic cancer [153,154].

Ferroptosis not only affects tumor cells but also is closely related to the function of immune cells. For instance, CD8+ T cells downregulate the expression of SLC3A2 and SLC7A11 in tumor cells by releasing interferon-γ (IFN-γ), thereby promoting the occurrence of ferroptosis [70]. This mechanism is particularly important in immune checkpoint blockade therapy, as enhanced ferroptosis can significantly improve T cell-mediated anti-tumor immune responses [70,153]. In addition, ferroptosis can induce immunogenic cell death (ICD), activate dendritic cells (DCs) and T cells, and further strengthen immune responses [155,156]. Additionally, the synergistic effect between ferroptosis and cuproptosis has been proven to significantly enhance anti-tumor immune responses, providing a new perspective for cancer treatment [153].

Cuproptosis not only directly kills tumor cells but also activates the immune system by releasing DAMPs and promoting the maturation of antigen-presenting cells. Specifically, cuproptosis-induced endoplasmic reticulum stress and the release of DAMPs can trigger immunogenic cell death (ICD), thereby enhancing immune responses in the TME. In addition, cuproptosis can mobilize cytotoxic T lymphocytes (CTLs) and M1-type macrophages in the TME, further strengthening anti-tumor immunity [157].

Copper ionophores such as Elesclomol (ES) can specifically transport copper ions into the mitochondria of tumor cells, thereby inducing cuproptosis. To improve the stability and tumor targeting of ES, researchers have developed various nano-drug delivery systems, such as ES@CuO nanoparticles and TSF@ES-Cu nanoparticles, which have significantly enhanced the efficacy of cuproptosis and immunotherapy [158,159].

The combination therapy of cuproptosis and immune checkpoint inhibitors (such as PD-1/PD-L1 inhibitors) has shown significant anti-tumor efficacy. For example, the combined use of NP@ESCu nanoparticles and αPD-L1 antibodies not only induces cuproptosis but also significantly enhances the efficacy of immune checkpoint inhibitors [107]. In addition, the combined therapy of TSF@ES-Cu nanoparticles and αPD-L1 antibodies has also demonstrated higher anti-tumor effects than the use of either therapy alone [159]. Currently, clinical trials verifying the synergistic effect of cuproptosis and immunotherapy mainly include:NCT03034135 Safety, Tolerability and Efficacy of Disulfiram and Copper Gluconate in Recurrent Glioblastoma (Phase II). NCT00088088 STA-4783 in Combination With Paclitaxel and Carboplatin for the Treatment of Chemotherapy-Naive Patients With Stage IIIB/IV Non-Small Cell Lung Cancer (NSCLC) (Phase II). With the advancement and exploration of research on cuproptosis-related nanomaterials, an increasing number of cuproptosis-associated drugs will be combined with immunotherapy in clinical trials, emerging as a novel strategy for tumor treatment.

**Figure 7.**
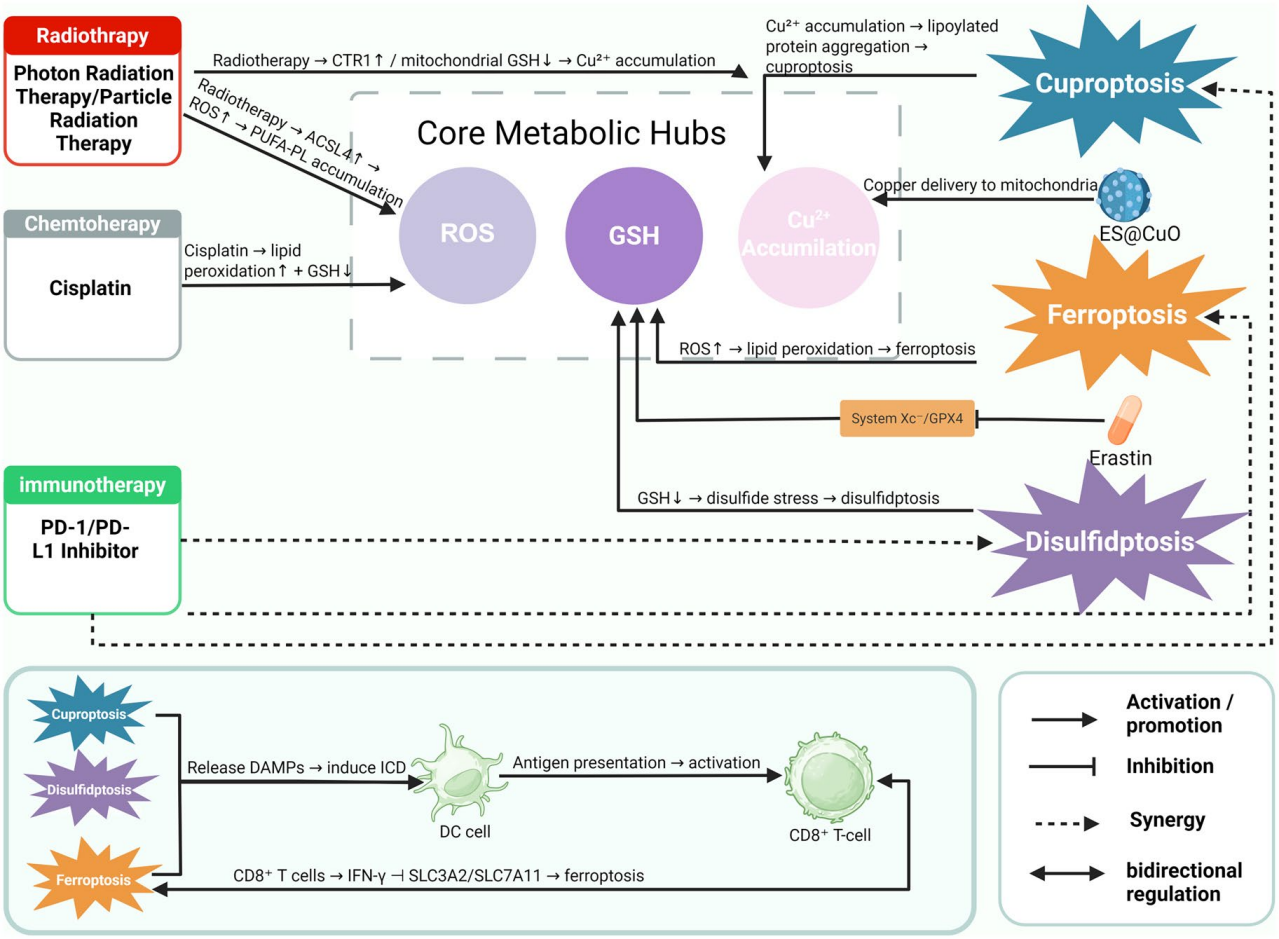
The figure themed ‘Therapeutic Input → Metabolic Hub → Cell Death → Immune Effect’ provides an analysis of the underlying network. In the upper-left section, radiotherapy is shown to connect to the metabolic hub through two distinct pathways. The middle section illustrates how chemotherapy enhances lipid peroxidation while simultaneously reducing GSH levels. In the lower-left section, immunotherapy is depicted as synergizing with metabolic cell death on the right side. The metabolic hub, consisting of ROS, GSH, and Cu^2+^, plays a crucial role in signal relay. The right side of the figure details three distinct modes of cell death: Cuproptosis (associated with Cu^2+^ and ES@CuO), Ferroptosis (triggered by ROS and Erastin), and Disulfidptosis (resulting from GSH depletion). At the bottom, it is illustrated that these modes of cell death release DAMPs that activate DCs and subsequently CD8^+^ T cells, leading to the production of interferon-gamma (IFN-γ) and a decrease in SLC3A2/SLC7A11, which promotes ferroptosis, thereby establishing a bidirectional regulatory mechanism.

Disulfidptosis can enhance anti-tumor immune responses by inducing immunogenic cell death, releasing a large amount of cellular contents and inflammatory factors, and activating dendritic cells and cytotoxic T cells [160]. Disulfidptosis can reshape the immunosuppressive TME, promote the infiltration of CD8+ T cells, and reduce the number of immunosuppressive cells (such as Tregs and M2 macrophages) [82,160]. This alteration of the microenvironment helps convert ‘cold tumors’ into ‘hot tumors’, thereby improving the response rate to immunotherapy [83,84]. Studies have shown that disulfidptosis exhibits a significant synergistic effect with immune checkpoint inhibitors (ICIs). For example, in bladder cancer and breast cancer, the combined use of disulfidptosis inducers and PD-1/PD-L1 inhibitors has significantly inhibited tumor growth and prolonged survival [83,160](Figure 7).

Currently, there is a lack of research on the relationship between lysozincrosis, alkaliptosis, and immunotherapy. These two types of metabolic cell death hold great potential in combination with immunotherapy. In the future, studies can be conducted on altering zinc distribution in cancer cells to explore how regulating zinc homeostasis can inhibit cancer cell growth or induce their death. Based on the sensitivity of tumor cells to pH, future research can focus on the massive release of DAMPs caused by alkaliptosis, aiming to investigate how alkaliptosis enhances anti-tumor immune responses by activating the immune system.

Current research primarily focuses on the synergistic effects between metabolic cell death and traditional treatment modalities, as evidenced by findings in ferroptosis and cuproptosis. However, there exists a research gap regarding disulfidptosis, lysozincrosis, and alkaliptosis. Future efforts should aim to address these gaps, optimize the combined administration strategies of metabolic cell death activators with radiotherapy and chemotherapy, and develop more effective and low-resistance next-generation combination therapies for patients with HNC.

Based on evidence indicating that single-target strategies seldom achieve durable control, and that combinatorial regimens informed by tumor evolution are preferable [85], we advocate for positioning metabolic cell-death programs—namely ferroptosis, cuproptosis, and disulfidptosis—as adjuvants in immunotherapy for HNC. Radiotherapy and platinum-based chemotherapy modify redox and metal homeostasis while increasing ROS, thereby metabolically priming tumors for lipid peroxidation and mitochondrial lipoyl stress. Aligning this priming with the induction of ferroptosis or cuproptosis could enhance antigen release and immunogenic cell death, facilitate dendritic cell cross-priming, and sensitize tumors to PD-1 or PD-L1 blockade. To translate this rationale into practical application, we propose a translational sequence utilizing full-width numbering: (1) schedule induction following radiotherapy or chemoradiotherapy when redox priming is at its peak, while minimizing lymphocyte exposure; (2) employ biomarker guidance based on ACSL4 activity, PUFA-phospholipid content, glutathione or GPX4 status, copper-handling metrics, and interferon-γor CD8 T-cell signatures to identify patients most likely to benefit from immune-permissive ferroptotic death; (3) utilize nano-delivery systems for copper or PUFA payloads to restrict toxicity to tumors; (4) incorporate early pharmacodynamic endpoints that evaluate synergy, including circulating tumor DNA (ctDNA) kinetics, lipid-peroxidation adducts, and T-cell-inflamed gene scores; (5) iterate dosing and sequencing through Bayesian adaptive designs to address evolutionary resistance. This framework operationalizes insights gained from the lengthy pursuit of RAS targeting into mechanism-based, immunologically coherent combinations for HNC [85].

## 8. Metabolic cell death therapy in HNC: Clinical translational barriers and innovative strategies

### 8.1. Tumor heterogeneity: the core barrier to precision targeting

HNC display high inter- and intratumoral heterogeneity, hindering metabolic cell death-based therapies. Tumor clonal populations vary in expressing key regulators of ferroptosis, cuproptosis, disulfidptosis, lysozincrosis, and alkaliptosis, leading to unequal sensitivity to these death triggers—for instance, HNC subclones with high GPX4 are intrinsically resistant to ferroptosis [64], while variability in FDX1 or TRPML1 affects susceptibility. This heterogeneity means uniform therapies may kill only sensitive fractions, sparing resistant clones and causing treatment failure or relapse. Innovative strategies are needed, such as precision medicine stratifying patients by metabolic profiles. Emerging studies use ferroptosis-related biomarkers (e.g. GPX4, SLC7A11, ACSL4) to predict responsiveness. Tailoring death inducers to specific molecular vulnerabilities or co-targeting multiple pathways may better address HNC heterogeneity and eliminate all malignant subpopulations.

### 8.2. Target specificity challenges: Microenvironment interference and off-target toxicity

Achieving tumor-specific metabolic cell death induction is complicated by TME interference and off-target risks. In HNC, cancer-associated fibroblasts (CAFs) and stromal components release factors blunting death inducer efficacy: CAFs secrete exosomal miR-522 to suppress lipid-peroxidation enzyme ALOX15, inhibiting ferroptosis, with chemotherapy paradoxically stimulating more such miR-522 release [86]. Cancer cell adaptation to hypoxia/acidosis drives progression—HNC often overexpresses carbonic anhydrase IX (CA9) to maintain alkaline intracellular pH. Off-target effects limit therapy: small-molecule inducers (e.g. GPX4 inhibitors, copper ionophores) may harm healthy cells, restricting the therapeutic window [87,88]. Innovative strategies include exploiting cancer-specific aberrations (e.g. activating TRPML1, upregulated in some cancers, to induce lysozincrosis selectively in cancer cells [137] and localizing treatment via nanoparticles or tumor enzyme-activated prodrugs. Overcoming these requires combining tumor-targeted delivery with TME modulation to prevent stromal rescue of cancer cells.

### 8.3. Delivery efficiency bottleneck

Efficient delivery of metabolic cell death inducers to HNC tumors remains a critical bottleneck. Many such compounds (e.g. lipophilic GPX4 inhibitors, alkaliptosis inducer JTC801) have suboptimal pharmacokinetics—poor water solubility and short half-lives lead to insufficient intratumoral concentrations [89]. Additionally, HNC’s dense stroma and irregular vasculature impede drug penetration, creating residual cancer cell ‘dead zones’. To address this, nanocarrier and prodrug technologies are explored: iron oxide nanoparticles (IONPs) ferry iron into cancer cells, catalyzing lipid peroxidation to induce ferroptosis [88], with engineered tumor-targeting ligands and controlled-release coatings improving accumulation and reducing systemic exposure. For cuproptosis, Cu^2+^ is delivered (intracellularly reduced to active Cu^+) to avoid premature redox reactions during transport [90]. Other approaches include synthetic carriers co-delivering metabolic inhibitors and death inducers (e.g. GLUT inhibitors with ferroptosis inducers). Developing such targeted delivery systems is essential to ensure therapeutically effective doses reach all tumor regions.

### 8.4. Therapy resistance and mechanism-driven reversal strategies

Even as metabolic cell death therapies circumvent traditional apoptosis resistance, tumors develop new resistance mechanisms, as cancer cells adapt metabolism/stress responses to select for cells upregulating defenses against ferroptosis, cuproptosis, etc. For ferroptosis, some cancer cells remain viable post-GPX4 inhibition via parallel rescue systems [86]. In HNC, pseudokinase TRIB3 forms a complex with β-catenin/TCF4 to repress pro-ferroptotic ALOXE3, inhibiting lipid peroxidation and promoting malignancy [48]. For cuproptosis, with limited research, potential resistance may arise from altered copper homeostasis (e.g. upregulated ATP7A/B efflux or downregulated CTR1 import), countered by combining copper ionophores with efflux inhibitors. Cancer cells may evade disulfidptosis via increased NADPH production or reduced SLC7A11, addressable by inhibiting compensatory metabolic routes. Lysozincrosis/alkaliptosis resistance is less documented but may involve modulated lysosomal ion channels/pH regulators. Ultimately, continual tumor molecular monitoring and nimble combination therapies are needed to outpace resistance evolution.

### 8.5. Clinical translational path: from biomarkers to personalized therapy

Translating metabolic cell death therapies for HNC to clinic needs biomarker-guided personalization. A crucial first step is identifying predictive biomarkers for response to specific death modalities. For ferroptosis, HNC studies highlight GPX4, SLC7A11, ACSL4, NCOA4, and composite signatures, with a ferroptosis-related gene risk score stratifying HNSCC patients by outcome [64]. Emerging models use cuproptosis/disulfidptosis gene sets/lncRNAs [92]. Clinical translation requires biomarker-enriched trials evaluating inducers in selected patients, possibly first in recurrent/refractory tumors with relevant biomarkers. Combinations with existing therapies are mechanistically rational. In summary, the clinical roadmap for metabolic cell death therapies in HNC involves: (1) discovery and validation of robust biomarkers and companion diagnostics; (2) development of targeted delivery systems to maximize tumor-specific action; (3) early-phase trials in biomarker-selected patient cohorts; and (4) eventually, incorporation into personalized treatment regimens, potentially as adjuvant or combination therapies for difficult-to-treat cancers. By embracing this biomarker-driven strategy, clinicians can leverage ferroptosis, cuproptosis, disulfidptosis, lysozincrosis, and alkaliptosis as new arrows in the quiver of individualized cancer therapy, aiming to improve outcomes in HNC beyond what conventional treatments can achieve.

## 9. Conclusion and prospects

Recent studies have generated considerable interest in the therapeutic roles and functions of metabolic cell death mechanisms, including ferroptosis, cuproptosis, and disulfidptosis, especially in the context of HNC. Ferroptosis, characterized by iron-dependent lipid peroxidation, displays a dual role in HNC, with key regulators such as ACSL4, FSP1, and PCBP1 being integral to this process [32,161–163]. Cuproptosis, induced by copper ions, inhibits cancer cell proliferation while promoting metastatic invasion; furthermore, copper ions may indirectly facilitate ferroptosis. Disulfidptosis, a process related to ferroptosis, has not been thoroughly investigated in the context of HNC cells, yet it is hypothesized to inhibit their growth. The exploration of metabolic cell death mechanisms provides new perspectives and potential biomarkers, as well as therapeutic targets for the diagnosis, treatment, and prognosis assessment of HNCs, including SLC7A11 and ACSL4.

Ferroptosis has emerged as the most extensively studied mechanism in HNC research, with inducers demonstrating the capacity to inhibit tumor growth and overcome drug resistance. Research on cuproptosis has focused on the diagnostic and prognostic significance of key genes, as well as the tumor-suppressive effects of related nanomaterials. Currently, there are no experimental reports addressing disulfidptosis in HNC, which has led to an increased emphasis on the significance of associated genes. Despite the progress made, several obstacles persist, including insufficient studies on lysosome-dependent cell death and alkaliptosis, a lack of comprehensive understanding of the molecular pathways involved in cuproptosis and disulfidptosis, and the need to clarify the interaction networks among various metabolic pathways of cell death. Furthermore, the early stages of clinical therapeutic drug development face challenges related to specificity, efficacy, safety, and clinical translation.

Future research on metabolic cell death in HNC should prioritize the coordinated advancement of in-depth molecular mechanism analysis, technological innovation, and clinical translation. The utilization of technologies such as single-cell transcriptomics will be essential for revealing the heterogeneity of metabolic death, investigating interaction networks and cross-regulatory mechanisms, and exploring the mechanisms of lysosomal dysfunction and alkaliptosis. Innovative therapeutic strategies targeting metabolic nodes should be developed, alongside the optimization of multi-target drug design and delivery. Clinical translation should involve stratifying diagnosis and treatment based on biomarkers, constructing machine learning models to predict therapeutic combinations, and validating safety through Phase III clinical trials. A deep integration of multi-omics technologies, nanomedicine, and artificial intelligence is necessary to overcome key bottlenecks, providing a new paradigm for the precision treatment of HNC and ultimately improving patient survival outcomes.

## Data Availability

Data sharing is not applicable to this article as no new data were created or analyzed in this study.

## References

[1] Bray F, Laversanne M, Sung H, et al. Global cancer statistics 2022: GLOBOCAN estimates of incidence and mortality worldwide for 36 cancers in 185 countries. CA Cancer J Clin. 2024;74(3):229–263. doi: 10.3322/caac.21834. 38572751

[2] Johnson DE, Burtness B, Leemans CR, et al. Head and neck squamous cell carcinoma. Nat Rev Dis Primers. 2020;6(1):92. doi: 10.1038/s41572-020-00224-3. 33243986 PMC7944998

[3] Kitamura N, Sento S, Yoshizawa Y, et al. Current trends and future prospects of molecular targeted therapy in head and neck squamous cell carcinoma. Int J Mol Sci. 2020;22(1):240. doi: 10.3390/ijms22010240. 33383632 PMC7795499

[4] Bray F, Ferlay J, Soerjomataram I, et al. Global cancer statistics 2018: GLOBOCAN estimates of incidence and mortality worldwide for 36 cancers in 185 countries. CA Cancer J Clin. 2018;68(6):394–424. doi: 10.3322/caac.21492. 30207593

[5] Cramer JD, Burtness B, Le QT, et al. The changing therapeutic landscape of head and neck cancer. Nat Rev Clin Oncol. 2019;16(11):669–683. doi: 10.1038/s41571-019-0227-z. 31189965

[6] Pulte D, Brenner H. Changes in survival in head and neck cancers in the late 20th and early 21st century: a period analysis. Oncologist. 2010;15(9):994–1001. doi: 10.1634/theoncologist.2009-0289. 20798198 PMC3228039

[7] Li ZZ, Zhou K, Wu Q, et al. Lymph node metastasis in cancer: clearing the clouds to see the dawn. Crit Rev Oncol Hematol. 2024;204:104536. doi: 10.1016/j.critrevonc.2024.104536. 39426554

[8] Green DR, Victor B. The pantheon of the fallen: why are there so many forms of cell death? Trends Cell Biol. 2012;22(11):555–556. doi: 10.1016/j.tcb.2012.08.008. 22995729 PMC3568685

[9] Dixon SJ, Lemberg KM, Lamprecht MR, et al. Ferroptosis: an iron-dependent form of nonapoptotic cell death. Cell. 2012;149(5):1060–1072. doi: 10.1016/j.cell.2012.03.042. 22632970 PMC3367386

[10] Zhang Y, Lu Y, Jin L. Iron metabolism and ferroptosis in physiological and pathological pregnancy. Int J Mol Sci. 2022;23(16):9395. doi: 10.3390/ijms23169395. 36012659 PMC9409111

[11] Lawson DM, Artymiuk PJ, Yewdall SJ, et al. Solving the structure of human H ferritin by genetically engineering intermolecular crystal contacts. Nature. 1991;349(6309):541–544. doi: 10.1038/349541a0. 1992356

[12] Mancias JD, Wang X, Gygi SP, et al. Quantitative proteomics identifies NCOA4 as the cargo receptor mediating ferritinophagy. Nature. 2014;509(7498):105–109. doi: 10.1038/nature13148. 24695223 PMC4180099

[13] Harayama T, Riezman H. Understanding the diversity of membrane lipid composition. Nat Rev Mol Cell Biol. 2018;19(5):281–296. doi: 10.1038/nrm.2017.138. 29410529

[14] Jiang X, Stockwell BR, Conrad M. Ferroptosis: mechanisms, biology and role in disease. Nat Rev Mol Cell Biol. 2021;22(4):266–282. doi: 10.1038/s41580-020-00324-8. 33495651 PMC8142022

[15] Proneth B, Conrad M. Ferroptosis and necroinflammation, a yet poorly explored link. Cell Death Differ. 2019;26(1):14–24. doi: 10.1038/s41418-018-0173-9. 30082768 PMC6294786

[16] Shah R, Shchepinov MS, Pratt DA. Resolving the role of lipoxygenases in the initiation and execution of ferroptosis. ACS Cent Sci. 2018;4(3):387–396. doi: 10.1021/acscentsci.7b00589. 29632885 PMC5879472

[17] Chen X, Kang R, Tang D. Ferroptosis by lipid peroxidation: the tip of the iceberg? Front Cell Dev Biol. 2021;9:646890. doi: 10.3389/fcell.2021.646890. 33842471 PMC8027319

[18] Su LJ, Zhang JH, Gomez H, et al. Reactive oxygen species-induced lipid peroxidation in apoptosis, autophagy, and ferroptosis. Oxid Med Cell Longev. 2019;2019:5080843–5080813. doi: 10.1155/2019/5080843. 31737171 PMC6815535

[19] He L, He T, Farrar S, et al. Antioxidants maintain cellular redox homeostasis by elimination of reactive oxygen species. Cell Physiol Biochem. 2017;44(2):532–553. doi: 10.1159/000485089. 29145191

[20] Matoušková P, Hanousková B, Skálová L. MicroRNAs as potential regulators of glutathione peroxidases expression and their role in obesity and related pathologies. Int J Mol Sci. 2018;19(4):1199. doi: 10.3390/ijms19041199. PMC5979329 29662007

[21] Bela K, Horváth E, Gallé Á, et al. Plant glutathione peroxidases: emerging role of the antioxidant enzymes in plant development and stress responses. J Plant Physiol. 2015;176:192–201. doi: 10.1016/j.jplph.2014.12.014. 25638402

[22] Brigelius-Flohé R. Glutathione peroxidases and redox-regulated transcription factors. Biol Chem. 2006;387(10–11):1329–1335. 17081103 10.1515/BC.2006.166

[23] Ursini F, Maiorino M, Valente M, et al. Purification from pig liver of a protein which protects liposomes and biomembranes from peroxidative degradation and exhibits glutathione peroxidase activity on phosphatidylcholine hydroperoxides. Biochim Biophys Acta. 1982;710(2):197–211. doi: 10.1016/0005-2760(82)90150-3. 7066358

[24] Seibt TM, Proneth B, Conrad M. Role of GPX4 in ferroptosis and its pharmacological implication. Free Radic Biol Med. 2019;133:144–152. doi: 10.1016/j.freeradbiomed.2018.09.014. 30219704

[25] Yang WS, SriRamaratnam R, Welsch ME, et al. Regulation of ferroptotic cancer cell death by GPX4. Cell. 2014;156(1–2):317–331. doi: 10.1016/j.cell.2013.12.010. 24439385 PMC4076414

[26] Zheng Y, Sun J, Luo Z, et al. Emerging mechanisms of lipid peroxidation in regulated cell death and its physiological implications. Cell Death Dis. 2024;15(11):859. doi: 10.1038/s41419-024-07244-x. 39587094 PMC11589755

[27] Shin CS, Mishra P, Watrous JD, et al. The glutamate/cystine xCT antiporter antagonizes glutamine metabolism and reduces nutrient flexibility. Nat Commun. 2017;8(1):15074. doi: 10.1038/ncomms15074. 28429737 PMC5413954

[28] Kim JW, Kim MJ, Han TH, et al. FSP1 confers ferroptosis resistance in KEAP1 mutant non-small cell lung carcinoma in NRF2-dependent and -independent manner. Cell Death Dis. 2023;14(8):567. doi: 10.1038/s41419-023-06070-x. 37633973 PMC10460413

[29] Xavier da Silva TN, Schulte C, Alves AN, et al. Molecular characterization of AIFM2/FSP1 inhibition by iFSP1-like molecules. Cell Death Dis. 2023;14(4):281. doi: 10.1038/s41419-023-05787-z. 37080964 PMC10119282

[30] Lee J, Roh JL. Unleashing ferroptosis in human cancers: targeting ferroptosis suppressor protein 1 for overcoming therapy resistance. Antioxidants. 2023;12(6):1218. doi: 10.3390/antiox12061218. PMC10295722 37371948

[31] Doll S, Freitas FP, Shah R, et al. FSP1 is a glutathione-independent ferroptosis suppressor. Nature. 2019;575(7784):693–698. doi: 10.1038/s41586-019-1707-0. 31634899

[32] Li W, Liang L, Liu S, et al. FSP1: a key regulator of ferroptosis. Trends Mol Med. 2023;29(9):753–764. doi: 10.1016/j.molmed.2023.05.013. 37357101

[33] Bersuker K, Hendricks JM, Li Z, et al. The CoQ oxidoreductase FSP1 acts parallel to GPX4 to inhibit ferroptosis. Nature. 2019;575(7784):688–692. doi: 10.1038/s41586-019-1705-2. 31634900 PMC6883167

[34] Anandhan A, Dodson M, Schmidlin CJ, et al. Breakdown of an ironclad defense system: the critical role of NRF2 in mediating ferroptosis. Cell Chem Biol. 2020;27(4):436–447. doi: 10.1016/j.chembiol.2020.03.011. 32275864 PMC7597851

[35] Roh JL. Targeting ferroptosis suppressor protein 1 in cancer therapy: implications and perspectives, with emphasis on head and neck cancer. Crit Rev Oncol Hematol. 2024;202:104440. doi: 10.1016/j.critrevonc.2024.104440. 38986728

[36] Yu R, Hang Y, Tsai HI, et al. Iron metabolism: backfire of cancer cell stemness and therapeutic modalities. Cancer Cell Int. 2024;24(1):157. doi: 10.1186/s12935-024-03329-x. 38704599 PMC11070091

[37] Badran O, Cohen I, Bar-Sela G. The impact of iron on cancer-related immune functions in oncology: molecular mechanisms and clinical evidence. Cancers. 2024;16(24):4156. doi: 10.3390/cancers16244156. 39766056 PMC11674619

[38] Tang D, Kepp O, Kroemer G. Ferroptosis becomes immunogenic: implications for anticancer treatments. Oncoimmunology. 2020;10(1):1862949. doi: 10.1080/2162402X.2020.1862949. 33457081 PMC7781761

[39] Zhu W, Liu X, Yang L, et al. Ferroptosis and tumor immunity: in perspective of the major cell components in the tumor microenvironment. Eur J Pharmacol. 2023;961:176124. doi: 10.1016/j.ejphar.2023.176124. 37925133

[40] Serrano-Gomez SJ, Maziveyi M, Alahari SK. Regulation of epithelial-mesenchymal transition through epigenetic and post-translational modifications. Mol Cancer. 2016;15(1):18. doi: 10.1186/s12943-016-0502-x. 26905733 PMC4765192

[41] Yi J, Zhu J, Wu J, et al. Oncogenic activation of PI3K-AKT-mTOR signaling suppresses ferroptosis via SREBP-mediated lipogenesis. Proc Natl Acad Sci USA. 2020;117(49):31189–31197. doi: 10.1073/pnas.2017152117. 33229547 PMC7733797

[42] Chen R, Zhu S, Zhao R, et al. Targeting ferroptosis as a potential strategy to overcome the resistance of cisplatin in oral squamous cell carcinoma. Front Pharmacol. 2024;15:1402514. doi: 10.3389/fphar.2024.1402514. 38711989 PMC11071065

[43] Teng Y, Gao L, Mäkitie AA, et al. Iron, ferroptosis, and head and neck cancer. Int J Mol Sci. 2023;24(20):15127. doi: 10.3390/ijms242015127. PMC10606477 37894808

[44] Li B, Yang L, Peng X, et al. Emerging mechanisms and applications of ferroptosis in the treatment of resistant cancers. Biomed Pharmacother. 2020;130:110710. doi: 10.1016/j.biopha.2020.110710. 33568263

[45] Qiu Y, Su Y, Sai W, et al. Research progress on ferroptosis in head and neck squamous cell carcinoma. J Mol Histol. 2025;56(2):109. doi: 10.1007/s10735-025-10381-y. 40095205

[46] Wu Y, Chen X, Chen Z, et al. Targeting ferroptosis in tumors: novel marine-derived compounds as regulators of lipid peroxidation and GPX4 signaling. Mar Drugs. 2025;23(6):258. doi: 10.3390/md23060258. 40559667 PMC12194754

[47] Jiang M, Qiao M, Zhao C, et al. Targeting ferroptosis for cancer therapy: exploring novel strategies from its mechanisms and role in cancers. Transl Lung Cancer Res. 2020;9(4):1569–1584. doi: 10.21037/tlcr-20-341. 32953528 PMC7481593

[48] Chen L, Lin W, Zhang H, et al. TRIB3 promotes malignancy of head and neck squamous cell carcinoma via inhibiting ferroptosis. Cell Death Dis. 2024;15(3):178. doi: 10.1038/s41419-024-06472-5. 38429254 PMC10907716

[49] Zhou Q, Meng Y, Li D, et al. Ferroptosis in cancer: from molecular mechanisms to therapeutic strategies. Signal Transduct Target Ther. 2024;9(1):55. doi: 10.1038/s41392-024-01769-5. 38453898 PMC10920854

[50] Lei G, Zhuang L, Gan B. Targeting ferroptosis as a vulnerability in cancer. Nat Rev Cancer. 2022;22(7):381–396. doi: 10.1038/s41568-022-00459-0. 35338310 PMC10243716

[51] Xu L, Zhou C, Liang Y, et al. Epigenetic modifications in the accumulation and function of myeloid-derived suppressor cells. Front Immunol. 2022;13:1016870. doi: 10.3389/fimmu.2022.1016870. 36439186 PMC9691837

[52] Chung CH, Lin CY, Chen CY, et al. Ferroptosis signature shapes the immune profiles to enhance the response to immune checkpoint inhibitors in head and neck cancer. Adv Sci. 2023;10(15):e2204514. doi: 10.1002/advs.202204514. PMC10214241 37026630

[53] Gao W, Wang X, Zhou Y, et al. Autophagy, ferroptosis, pyroptosis, and necroptosis in tumor immunotherapy. Signal Transduct Target Ther. 2022;7(1):196. 35725836 10.1038/s41392-022-01046-3 PMC9208265

[54] Ren Y, Mao X, Xu H, et al. Ferroptosis and EMT: key targets for combating cancer progression and therapy resistance. Cell Mol Life Sci. 2023;80(9):263. doi: 10.1007/s00018-023-04907-4. 37598126 PMC10439860

[55] Zhai Q, Wang Z, Tang H, et al. Identification of ferroptosis-associated tumor antigens as the potential targets to prevent head and neck squamous cell carcinoma. Genes Dis. 2024;11(6):101212. doi: 10.1016/j.gendis.2024.101212. 39286654 PMC11403004

[56] Chu B, Kon N, Chen D, et al. ALOX12 is required for p53-mediated tumour suppression through a distinct ferroptosis pathway. Nat Cell Biol. 2019;21(5):579–591. doi: 10.1038/s41556-019-0305-6. 30962574 PMC6624840

[57] Wang L, Chen X, Yan C. Ferroptosis: an emerging therapeutic opportunity for cancer. Genes Dis. 2022;9(2):334–346. doi: 10.1016/j.gendis.2020.09.005. 35224150 PMC8843872

[58] Yang J, Gu Z. Ferroptosis in head and neck squamous cell carcinoma: from pathogenesis to treatment. Front Pharmacol. 2024;15:1283465. doi: 10.3389/fphar.2024.1283465. 38313306 PMC10834699

[59] Gan B. How erastin assassinates cells by ferroptosis revealed. Protein Cell. 2023;14(2):84–86. 36929006 10.1093/procel/pwac007 PMC10019563

[60] Sinha BK, Murphy C, Brown SM, et al. Mechanisms of cell death induced by erastin in human ovarian tumor cells. Int J Mol Sci. 2024;25(16):8666. doi: 10.3390/ijms25168666 PMC11355013 39201357

[61] Wang L, Wang C, Li X, et al. Melatonin and erastin emerge synergistic anti-tumor effects on oral squamous cell carcinoma by inducing apoptosis, ferroptosis, and inhibiting autophagy through promoting ROS. Cell Mol Biol Lett. 2023;28(1):36. doi: 10.1186/s11658-023-00449-6. 37131152 PMC10155313

[62] Wu X, Wu J. A polo-like kinase 1 inhibitor enhances erastin sensitivity in head and neck squamous cell carcinoma cells in vitro. Cancer Chemother Pharmacol. 2024;94(2):183–195. doi: 10.1007/s00280-024-04654-8. 38536443 PMC11390781

[63] Roh JL, Kim EH, Jang HJ, et al. Induction of ferroptotic cell death for overcoming cisplatin resistance of head and neck cancer. Cancer Lett. 2016;381(1):96–103. doi: 10.1016/j.canlet.2016.07.035. 27477897

[64] Lee J, Seo Y, Roh JL. Emerging therapeutic strategies targeting GPX4-mediated ferroptosis in head and neck cancer. Int J Mol Sci. 2025;26(13):6452. doi: 10.3390/ijms26136452. PMC12250494 40650229

[65] Costa I, Barbosa DJ, Benfeito S, et al. Molecular mechanisms of ferroptosis and their involvement in brain diseases. Pharmacol Ther. 2023;244:108373. doi: 10.1016/j.pharmthera.2023.108373. 36894028

[66] Ye J, Jiang X, Dong Z, et al. Low-concentration PTX and RSL3 inhibits tumor cell growth synergistically by inducing ferroptosis in mutant p53 hypopharyngeal squamous carcinoma. Cancer Manag Res. 2019;11:9783–9792. doi: 10.2147/CMAR.S217944. 31819616 PMC6876222

[67] Wang Z, Li M, Liu Y, et al. Dihydroartemisinin triggers ferroptosis in primary liver cancer cells by promoting and unfolded protein response‑induced upregulation of CHAC1 expression. Oncol Rep. 2021;46(5):240. doi: 10.3892/or.2021.8191. PMC8485000 34558645

[68] Lin R, Zhang Z, Chen L, et al. Dihydroartemisinin (DHA) induces ferroptosis and causes cell cycle arrest in head and neck carcinoma cells. Cancer Lett. 2016;381(1):165–175. doi: 10.1016/j.canlet.2016.07.033. 27477901

[69] Noh JK, Lee MK, Lee Y, et al. Targeting ferroptosis for improved radiotherapy outcomes in HPV-negative head and neck squamous cell carcinoma. Mol Oncol. 2025;19(2):540–557. doi: 10.1002/1878-0261.13720. 39297393 PMC11792990

[70] Wang W, Green M, Choi JE, et al. CD8(+) T cells regulate tumour ferroptosis during cancer immunotherapy. Nature. 2019;569(7755):270–274. doi: 10.1038/s41586-019-1170-y. 31043744 PMC6533917

[71] Piccolo S, Dupont S, Cordenonsi M. The biology of YAP/TAZ: hippo signaling and beyond. Physiol Rev. 2014;94(4):1287–1312. doi: 10.1152/physrev.00005.2014. 25287865

[72] Chen J, Jiang Y, Shi H, et al. The molecular mechanisms of copper metabolism and its roles in human diseases. Pflugers Arch. 2020;472(10):1415–1429. doi: 10.1007/s00424-020-02412-2. 32506322

[73] Xue Q, Kang R, Klionsky DJ, et al. Copper metabolism in cell death and autophagy. Autophagy. 2023;19(8):2175–2195. doi: 10.1080/15548627.2023.2200554. 37055935 PMC10351475

[74] Chen L, Min J, Wang F. Copper homeostasis and cuproptosis in health and disease. Signal Transduct Target Ther. 2022;7(1):378. doi: 10.1038/s41392-022-01229-y. 36414625 PMC9681860

[75] Itoh S, Kim HW, Nakagawa O, et al. Novel role of antioxidant-1 (Atox1) as a copper-dependent transcription factor involved in cell proliferation. J Biol Chem. 2008;283(14):9157–9167. doi: 10.1074/jbc.M709463200. 18245776 PMC2431038

[76] Tsvetkov P, Coy S, Petrova B, et al. Copper induces cell death by targeting lipoylated TCA cycle proteins. Science. 2022;375(6586):1254–1261. doi: 10.1126/science.abf0529. 35298263 PMC9273333

[77] Kong R, Sun G. Targeting copper metabolism: a promising strategy for cancer treatment. Front Pharmacol. 2023;14:1203447. doi: 10.3389/fphar.2023.1203447. 37564178 PMC10411510

[78] Cong Y, Li N, Zhang Z, et al. Cuproptosis: molecular mechanisms, cancer prognosis, and therapeutic applications. J Transl Med. 2025;23(1):104. doi: 10.1186/s12967-025-06121-1. 39844182 PMC11752808

[79] Li Y, Ma J, Wang R, et al. Zinc transporter 1 functions in copper uptake and cuproptosis. Cell Metab. 2024;36(9):2118–2129 e6. doi: 10.1016/j.cmet.2024.07.009. 39111308

[80] Gerner MC, Bileck A, Janker L, et al. Packed red blood cells inhibit T-cell activation via ROS-dependent signaling pathways. J Biol Chem. 2021;296:100487. doi: 10.1016/j.jbc.2021.100487. 33676898 PMC8042437

[81] Li J, Chen S, Liao Y, et al. Arecoline is associated with inhibition of cuproptosis and proliferation of cancer-associated fibroblasts in oral squamous cell carcinoma: a potential mechanism for tumor metastasis. Front Oncol. 2022;12:925743. doi: 10.3389/fonc.2022.925743. 35875097 PMC9303015

[82] Xiao Y, Lin H, Li J, et al. Disulfidptosis-related prognostic signature correlates with immunotherapy response in colorectal cancer. Sci Rep. 2024;14(1):81. doi: 10.1038/s41598-023-49954-w. 38168553 PMC10762008

[83] Wang K, Li L, Liang G, et al. Sonodynamic activated nanoparticles with Glut1 inhibitor and cystine-containing polymer stimulate disulfidptosis for improved immunotherapy in bladder cancer. Biomaterials. 2025;319:123178. doi: 10.1016/j.biomaterials.2025.123178. 39978048

[84] Chen G, Zhang G, Zhu Y, et al. Identifying disulfidptosis subtypes in hepatocellular carcinoma through machine learning and preliminary exploration of its connection with immunotherapy. Cancer Cell Int. 2024;24(1):194. doi: 10.1186/s12935-024-03387-1. 38831301 PMC11149214

[85] Weinberg RA. It took a long, long time: ras and the race to cure cancer. Cell. 2024;187(21):6123. doi: 10.1016/j.cell.2024.09.010. 39303714

[86] Chen C, Liu J, Lin X, et al. Crosstalk between cancer-associated fibroblasts and regulated cell death in tumors: insights into apoptosis, autophagy, ferroptosis, and pyroptosis. Cell Death Discov. 2024;10(1):189. doi: 10.1038/s41420-024-01958-9. 38649701 PMC11035635

[87] Zheng G, Peng C, Jia X, et al. ZEB1 transcriptionally regulated carbonic anhydrase 9 mediates the chemoresistance of tongue cancer via maintaining intracellular pH. Mol Cancer. 2015;14(1):84. doi: 10.1186/s12943-015-0357-6. 25890268 PMC4404088

[88] Sant’Angelo D, Descamps G, Lecomte V, et al. Therapeutic approaches with iron oxide nanoparticles to induce ferroptosis and overcome radioresistance in cancers. Pharmaceuticals. 2025;18(3):325. doi: 10.3390/ph18030325. PMC11945075 40143107

[89] Zhang Y, Tan H, Daniels JD, et al. Imidazole ketone erastin induces ferroptosis and slows tumor growth in a mouse lymphoma model. Cell Chem Biol. 2019;26(5):623–633.e9. doi: 10.1016/j.chembiol.2019.01.008. 30799221 PMC6525071

[90] Feng Q, Huo C, Wang M, et al. Research progress on cuproptosis in cancer. Front Pharmacol. 2024;15:1290592. doi: 10.3389/fphar.2024.1290592. 38357312 PMC10864558

[91] Wang H, Yang Z, He X, et al. Cuproptosis related gene PDHB is identified as a biomarker inversely associated with the progression of clear cell renal cell carcinoma. BMC Cancer. 2023;23(1):804. doi: 10.1186/s12885-023-11324-0. 37641032 PMC10464351

[92] Liao H, He B. Predictive value of cuproptosis and disulfidptosis-related lncRNA in head and neck squamous cell carcinoma prognosis and treatment. Heliyon. 2024;10(18):e37996. doi: 10.1016/j.heliyon.2024.e37996. 39323825 PMC11422553

[93] Stockwell BR, Friedmann Angeli JP, Bayir H, et al. Ferroptosis: a regulated cell death nexus linking metabolism, redox biology, and disease. Cell. 2017;171(2):273–285. doi: 10.1016/j.cell.2017.09.021. 28985560 PMC5685180

[94] Lv J, Hou B, Song J, et al. The relationship between ferroptosis and diseases. J Multidiscip Healthc. 2022;15:2261–2275. doi: 10.2147/JMDH.S382643. 36225859 PMC9549801

[95] Wang W, Lu K, Jiang X, et al. Ferroptosis inducers enhanced cuproptosis induced by copper ionophores in primary liver cancer. J Exp Clin Cancer Res. 2023;42(1):142. doi: 10.1186/s13046-023-02720-2. 37277863 PMC10242978

[96] Xue Q, Yan D, Chen X, et al. Copper-dependent autophagic degradation of GPX4 drives ferroptosis. Autophagy. 2023;19(7):1982–1996. doi: 10.1080/15548627.2023.2165323. 36622894 PMC10283421

[97] Shimada K, Reznik E, Stokes ME, et al. Copper-binding small molecule induces oxidative stress and cell-cycle arrest in glioblastoma-patient-derived cells. Cell Chem Biol. 2018;25(5):585–594.e7. doi: 10.1016/j.chembiol.2018.02.010. 29576531 PMC5959763

[98] Liu J, Peng Y, Wei W. Cell cycle on the crossroad of tumorigenesis and cancer therapy. Trends Cell Biol. 2022;32(1):30–44. doi: 10.1016/j.tcb.2021.07.001. 34304958 PMC8688170

[99] Feng Y, Yang Z, Wang J, et al. Cuproptosis: unveiling a new frontier in cancer biology and therapeutics. Cell Commun Signal. 2024;22(1):249. doi: 10.1186/s12964-024-01625-7. 38693584 PMC11064406

[100] Liu X, Cheng W, Li H, et al. Identification and validation of cuproptosis-related LncRNA signatures as a novel prognostic model for head and neck squamous cell cancer. Cancer Cell Int. 2022;22(1):345. doi: 10.1186/s12935-022-02762-0. 36369058 PMC9652850

[101] Zhang Z, Wu Y, Wang SY, et al. Copper-related cell death and the role of copper in head and neck squamous cell carcinoma and therapeutic strategies. Adv Ther. 2024;7(5):2300198. doi: 10.1002/adtp.202300198.

[102] Wang M, Zheng L, Ma S, et al. Cuproptosis: emerging biomarkers and potential therapeutics in cancers. Front Oncol. 2023;13:1288504. doi: 10.3389/fonc.2023.1288504. 38023234 PMC10662309

[103] Ge EJ, Bush AI, Casini A, et al. Connecting copper and cancer: from transition metal signalling to metalloplasia. Nat Rev Cancer. 2022;22(2):102–113. doi: 10.1038/s41568-021-00417-2. 34764459 PMC8810673

[104] Zhang C, Huang T, Li L. Targeting cuproptosis for cancer therapy: mechanistic insights and clinical perspectives. J Hematol Oncol. 2024;17(1):68. doi: 10.1186/s13045-024-01589-8. 39152464 PMC11328505

[105] Chen S, Sun L, Koya K, et al. Syntheses and antitumor activities of N’1,N’3-dialkyl-N’1,N’3-di-(alkylcarbonothioyl) malonohydrazide: the discovery of elesclomol. Bioorg Med Chem Lett. 2013;23(18):5070–5076. 23937981 10.1016/j.bmcl.2013.07.032

[106] Lu C, Li X, Ren Y, et al. Disulfiram: a novel repurposed drug for cancer therapy. Cancer Chemother Pharmacol. 2021;87(2):159–172. doi: 10.1007/s00280-020-04216-8. 33426580

[107] Guo B, Yang F, Zhang L, et al. Cuproptosis induced by ROS responsive nanoparticles with elesclomol and copper combined with αPD-L1 for enhanced cancer immunotherapy. Adv Mater. 2023;35(22):e2212267. 36916030 10.1002/adma.202212267

[108] Zhao S, Chen S, Liu W, et al. Integrated machine learning and bioinformatic analyses used to construct a copper-induced cell death-related classifier for prognosis and immunotherapeutic response of hepatocellular carcinoma patients. Front Pharmacol. 2023;14:1188725. doi: 10.3389/fphar.2023.1188725. 37266152 PMC10229845

[109] Yang Z, Su W, Wei X, et al. Hypoxia inducible factor-1α drives cancer resistance to cuproptosis. Cancer Cell. 2025;43(5):937–954.e9. doi: 10.1016/j.ccell.2025.02.015. 40054467

[110] Li ZZ, Liu Y, Zhou K, et al. ORL@Cu-MOF boost cuproptosis and suppress fatty acid metabolism for cancer lymph node metastasis synergistic therapy. Adv Sci. 2025;12(35):e02154. 10.1002/advs.202502154 PMC12463119 40548889

[111] Wang S, Liu Y, Su M, et al. Near-infrared activatable copper nanoplatforms synergize with the 5-azacytidine prodrug to potentiate cuproptosis. Angew Chem Int Ed Engl. 2024;63(52):e202411609. doi: 10.1002/anie.202411609. 39400411

[112] Liu X, Nie L, Zhang Y, et al. Actin cytoskeleton vulnerability to disulfide stress mediates disulfidptosis. Nat Cell Biol. 2023;25(3):404–414. doi: 10.1038/s41556-023-01091-2. 36747082 PMC10027392

[113] Wang J, Chen J, Fan K, et al. Inhibition of endoplasmic reticulum stress cooperates with SLC7A11 to promote disulfidptosis and suppress tumor growth upon glucose limitation. Adv Sci. 2025;12(7):e2408789. doi: 10.1002/advs.202408789. PMC11831432 39739602

[114] Victor P, Sarada D, Ramkumar KM. Crosstalk between endoplasmic reticulum stress and oxidative stress: focus on protein disulfide isomerase and endoplasmic reticulum oxidase 1. Eur J Pharmacol. 2021;892:173749. doi: 10.1016/j.ejphar.2020.173749. 33245896

[115] Hetz C, Zhang K, Kaufman RJ. Mechanisms, regulation and functions of the unfolded protein response. Nat Rev Mol Cell Biol. 2020;21(8):421–438. doi: 10.1038/s41580-020-0250-z. 32457508 PMC8867924

[116] Qian S, Chen G, Li R, et al. Disulfide stress and its role in cardiovascular diseases. Redox Biol. 2024;75:103297. doi: 10.1016/j.redox.2024.103297. 39127015 PMC11364009

[117] Yao HF, Ge J, Chen J, et al. CASC8 activates the pentose phosphate pathway to inhibit disulfidptosis in pancreatic ductal adenocarcinoma though the c-Myc-GLUT1 axis. J Exp Clin Cancer Res. 2025;44(1):26. doi: 10.1186/s13046-025-03295-w. 39865281 PMC11771065

[118] Mailloux RJ. Protein S-glutathionylation reactions as a global inhibitor of cell metabolism for the desensitization of hydrogen peroxide signals. Redox Biol. 2020;32:101472. doi: 10.1016/j.redox.2020.101472. 32171726 PMC7076094

[119] Rashdan NA, Shrestha B, Pattillo CB. S-glutathionylation, friend or foe in cardiovascular health and disease. Redox Biol. 2020;37:101693. doi: 10.1016/j.redox.2020.101693. 32912836 PMC7767732

[120] Chai YC, Mieyal JJ. Glutathione and glutaredoxin-key players in cellular redox homeostasis and signaling. Antioxidants. 2023;12(8):1553. doi: 10.3390/antiox12081553. PMC10451691 37627548

[121] Seitz R, Tümen D, Kunst C, et al. Exploring the thioredoxin system as a therapeutic target in cancer: mechanisms and implications. Antioxidants. 2024;13(9):1078. doi: 10.3390/antiox13091078. PMC11428833 39334737

[122] Xiao F, Li HL, Yang B, et al. Disulfidptosis: a new type of cell death. Apoptosis. 2024;29(9–10):1309–1329. doi: 10.1007/s10495-024-01989-8. 38886311 PMC11416406

[123] Chen H, Cao L, Han K, et al. Patulin disrupts SLC7A11-cystine-cysteine-GSH antioxidant system and promotes renal cell ferroptosis both *in vitro* and *in vivo*. Food Chem Toxicol. 2022;166:113255. doi: 10.1016/j.fct.2022.113255. 35772596

[124] Chen J, Ma B, Yang Y, et al. Disulfidptosis decoded: a journey through cell death mysteries, regulatory networks, disease paradigms and future directions. Biomark Res. 2024;12(1):45. doi: 10.1186/s40364-024-00593-x. 38685115 PMC11059647

[125] Adeva-Andany MM, Pérez-Felpete N, Fernández-Fernández C, et al. Liver glucose metabolism in humans. Biosci Rep. 2016;36(6):e00416. doi: 10.1042/BSR20160385. PMC5293555 27707936

[126] Li T, Song Y, Wei L, et al. Disulfidptosis: a novel cell death modality induced by actin cytoskeleton collapse and a promising target for cancer therapeutics. Cell Commun Signal. 2024;22(1):491. doi: 10.1186/s12964-024-01871-9. 39394612 PMC11470700

[127] Liu X, Zhuang L, Gan B. Disulfidptosis: disulfide stress-induced cell death. Trends Cell Biol. 2024;34(4):327–337. doi: 10.1016/j.tcb.2023.07.009. 37574347

[128] Yan Y, Teng H, Hang Q, et al. SLC7A11 expression level dictates differential responses to oxidative stress in cancer cells. Nat Commun. 2023;14(1):3673. doi: 10.1038/s41467-023-39401-9. 37339981 PMC10281978

[129] Wang J, Wang M, Wu S, et al. Tumor suppressor BAP1 suppresses disulfidptosis through the regulation of SLC7A11 and NADPH levels. Oncogenesis. 2024;13(1):31. doi: 10.1038/s41389-024-00535-0. 39266549 PMC11393423

[130] Gao L, Yang F, Tang D, et al. Mediation of PKM2-dependent glycolytic and non-glycolytic pathways by ENO2 in head and neck cancer development. J Exp Clin Cancer Res. 2023;42(1):1. doi: 10.1186/s13046-022-02574-0. 36588153 PMC9806895

[131] Vermot A, Petit-Härtlein I, Smith SME, et al. NADPH oxidases (NOX): an overview from discovery, molecular mechanisms to physiology and pathology. Antioxidants. 2021;10(6):890. doi: 10.3390/antiox10060890. PMC8228183 34205998

[132] Lee J, Roh JL. Unveiling therapeutic avenues targeting xCT in head and neck cancer. Cell Oncol. 2024;47(6):2019–2030. doi: 10.1007/s13402-024-00997-9. PMC12974016 39361147

[133] Sun XY, Xiao M, Fu M, et al. ALMS1-IT1: a key player in the novel disulfidptosis-related LncRNA prognostic signature for head and neck squamous cell carcinoma. Biomolecules. 2024;14(3):266. doi: 10.3390/biom14030266. 38540687 PMC10968447

[134] Qin H, Xu J, Yue Y, et al. Disulfidptosis-related gene signatures as prognostic biomarkers and predictors of immunotherapy response in HNSCC. Front Immunol. 2024;15:1456649. doi: 10.3389/fimmu.2024.1456649. 39896807 PMC11782277

[135] Zhu Y, Wang X, Feng L, et al. Intermetallics triggering pyroptosis and disulfidptosis in cancer cells promote anti-tumor immunity. Nat Commun. 2024;15(1):8696. doi: 10.1038/s41467-024-53135-2. 39379392 PMC11461493

[136] Shi ZZ, Tao H, Fan ZW, et al. Prognostic and immunological role of key genes of ferroptosis in pan-cancer. Front Cell Dev Biol. 2021;9:748925. doi: 10.3389/fcell.2021.748925. 34722530 PMC8548644

[137] Du W, Gu M, Hu M, et al. Lysosomal Zn(2+) release triggers rapid, mitochondria-mediated, non-apoptotic cell death in metastatic melanoma. Cell Rep. 2021;37(3):109848. doi: 10.1016/j.celrep.2021.109848. 34686351 PMC8559338

[138] Raudenská M, Balvan J, Masařík M. Cell death in head and neck cancer pathogenesis and treatment. Cell Death Dis. 2021;12(2):192. doi: 10.1038/s41419-021-03474-5. 33602906 PMC7893032

[139] Tang T, Yang ZY, Wang D, et al. The role of lysosomes in cancer development and progression. Cell Biosci. 2020;10(1):131. doi: 10.1186/s13578-020-00489-x. 33292489 PMC7677787

[140] Liu J, Kuang F, Kang R, et al. Alkaliptosis: a new weapon for cancer therapy. Cancer Gene Ther. 2020;27(5):267–269. doi: 10.1038/s41417-019-0134-6. 31467365

[141] Song X, Zhu S, Xie Y, et al. JTC801 induces pH-dependent death specifically in cancer cells and slows growth of tumors in mice. Gastroenterology. 2018;154(5):1480–1493. doi: 10.1053/j.gastro.2017.12.004. 29248440 PMC5880694

[142] Lin W, Wang C, Liu G, et al. SLC7A11/xCT in cancer: biological functions and therapeutic implications. Am J Cancer Res. 2020;10(10):3106–3126. 33163260 PMC7642655

[143] Mao C, Wang M, Zhuang L, et al. Metabolic cell death in cancer: ferroptosis, cuproptosis, disulfidptosis, and beyond. Protein Cell. 2024;15(9):642–660. doi: 10.1093/procel/pwae003. 38428031 PMC11365558

[144] Lee J, Roh JL. SLC7A11 as a gateway of metabolic perturbation and ferroptosis vulnerability in cancer. Antioxidants. 2022;11(12):2444. doi: 10.3390/antiox11122444. PMC9774303 36552652

[145] Zhu H, Zhao C, Zhu H, et al. The characteristics and functional significance of disulfidptosis-related genes in head and neck squamous cell carcinoma. Discov Oncol. 2024;15(1):739. doi: 10.1007/s12672-024-01629-2. 39625660 PMC11615178

[146] Xue H, Sun Q, Zhang H, et al. Disulfidptosis features and prognosis in head and neck squamous cell carcinoma patients: unveiling and validating the prognostic signature across cohorts. J Cancer Res Clin Oncol. 2024;150(3):156. doi: 10.1007/s00432-024-05691-9. 38526631 PMC10963584

[147] Feng Y, Li X, Yang B, et al. The role of ferroptosis in radiotherapy and combination therapy for head and neck squamous cell carcinoma. Oncol Rep. 2024;51(6):79. doi: 10.3892/or.2024.8738. PMC11056820 38639185

[148] Sun S, Shen J, Jiang J, et al. Targeting ferroptosis opens new avenues for the development of novel therapeutics. Signal Transduct Target Ther. 2023;8(1):372. 37735472 10.1038/s41392-023-01606-1 PMC10514338

[149] Reinema FV, Hudson N, Adema GJ, et al. MitoTam induces ferroptosis and increases radiosensitivity in head and neck cancer cells. Radiother Oncol. 2024;200:110503. doi: 10.1016/j.radonc.2024.110503. 39186982

[150] Lei G, Sun M, Cheng J, et al. Radiotherapy promotes cuproptosis and synergizes with cuproptosis inducers to overcome tumor radioresistance. Cancer Cell. 2025;43(6):1076–1092.e5. doi: 10.1016/j.ccell.2025.03.031. 40215978 PMC12151758

[151] Xu Y, Ge M, Xu Y, et al. Ferroptosis: a novel perspective on tumor immunotherapy. Front Immunol. 2025;16:1524711. doi: 10.3389/fimmu.2025.1524711. 40260246 PMC12009862

[152] Zheng P, Hu Z, Shen Y, et al. PSAT1 impairs ferroptosis and reduces immunotherapy efficacy via GPX4 hydroxylation. Nat Chem Biol. 2025;21(9):1420–1432. doi: 10.1038/s41589-025-01887-3. 40281343

[153] Li Y, Liu J, Chen Y, et al. Nanoparticles synergize ferroptosis and cuproptosis to potentiate cancer immunotherapy. Adv Sci 2024;11(23):e2310309. 10.1002/advs.202310309 PMC11187894 38477411

[154] Lin H, Zhu S, Chen Y, et al. Targeting cTRIP12 counteracts ferroptosis resistance and augments sensitivity to immunotherapy in pancreatic cancer. Drug Resist Updat. 2025;81:101240. doi: 10.1016/j.drup.2025.101240. 40154160

[155] Liu J, Zhan J, Zhang Y, et al. Ultrathin clay nanoparticles-mediated mutual reinforcement of ferroptosis and cancer immunotherapy. Adv Mater. 2024;36(9):e2309562. 37939375 10.1002/adma.202309562

[156] Zhang X, Zhang X, Fan Q, et al. Self-accelerated nanoregulators for positive feedback ferroptosis-immunotherapy. Small. 2025;21(14):e2408156. 40026025 10.1002/smll.202408156

[157] Li J, Zhang G, Sun Z, et al. Immunogenic cuproptosis in cancer immunotherapy via an in situ cuproptosis-inducing system. Biomaterials. 2025;319:123201. doi: 10.1016/j.biomaterials.2025.123201. 40020502

[158] Lu X, Chen X, Lin C, et al. Elesclomol loaded copper oxide nanoplatform triggers cuproptosis to enhance antitumor immunotherapy. Adv Sci. 2024;11(18):e2309984. doi: 10.1002/advs.202309984. PMC11095170 38430531

[159] Gao S, Ge H, Gao L, et al. Silk fibroin nanoparticles for enhanced cuproptosis and immunotherapy in pancreatic cancer treatment. Adv Sci. 2025;12(18):e2417676. doi: 10.1002/advs.202417676. PMC12079484 40091480

[160] Mohanty A, Mohapatra A, Yang W, et al. Programable prodrug nanomodulator targets tumor redox homeostasis imbalance to amplify disulfidptosis and immunogenic pyroptosis for breast tumor immunotherapy. Adv Healthc Mater. 2025;14(11):e2500272. 40109062 10.1002/adhm.202500272

[161] Wu YC, Huang CS, Hsieh MS, et al. Targeting of FSP1 regulates iron homeostasis in drug-tolerant persister head and neck cancer cells via lipid-metabolism-driven ferroptosis. Aging. 2024;16(1):627–647. doi: 10.18632/aging.205409. 38206305 PMC10817390

[162] Chen P, Wang D, Xiao T, et al. ACSL4 promotes ferroptosis and M1 macrophage polarization to regulate the tumorigenesis of nasopharyngeal carcinoma. Int Immunopharmacol. 2023;122:110629. doi: 10.1016/j.intimp.2023.110629. 37451020

[163] Lee J, You JH, Roh JL. Poly(rC)-binding protein 1 represses ferritinophagy-mediated ferroptosis in head and neck cancer. Redox Biol. 2022;51:102276. doi: 10.1016/j.redox.2022.102276. 35290903 PMC8921323

